# Ancient inversion polymorphisms are locally adaptive in a widespread butterfly species

**DOI:** 10.1126/sciadv.adv1982

**Published:** 2026-07-01

**Authors:** Fernando A. Seixas, Sarah Dendy, Shuzhe Guan, Neil Rosser, Nick Grishin, Neil Davies, Marysol Trujano-Ortega, Tanner C. Myers, Brian A. Counterman, Riccardo Papa, Lawrence E. Gilbert, W. Owen McMillan, James Mallet

**Affiliations:** ^1^ Department of Organismic and Evolutionary Biology, Harvard University, Cambridge, MA 02138, USA.; ^2^ Tree of Life Programme, Wellcome Sanger Institute, Hinxton CB10 1SA, UK.; ^3^ Department of Biology, University of Miami, Coral Gables, FL 33146, USA.; ^4^ Museum of Comparative Zoology, Harvard University, Cambridge, MA 02138, USA.; ^5^ Department of Biophysics, University of Texas Southwestern Medical Center, Dallas, TX 75390, USA.; ^6^ Department of Biochemistry, University of Texas Southwestern Medical Center, Dallas, TX 75390, USA.; ^7^ Gump South Pacific Research Station, UC Berkeley, BP 244, Maharepa 98728, French Polynesia.; ^8^ El Colegio de la Frontera Sur, Unidad Chetumal, Chetumal 77014, Mexico.; ^9^ Facultad de Ciencias, Departamento de Biología Evolutiva, UNAM, Ciudad de México 04510, Mexico.; ^10^ Department of Biological Sciences, Auburn University, Auburn, AL 36849, USA.; ^11^ Department of Biology, University of Puerto Rico at Río Piedras, San Juan 00925, Puerto Rico.; ^12^ Comprehensive Cancer Center, University of Puerto Rico, San Juan 00925, Puerto Rico.; ^13^ Molecular Sciences and Research Center, University of Puerto Rico, San Juan 00926, Puerto Rico.; ^14^ Dipartimento di Scienze Chimiche della Vita e della Sostenibilità Ambientale, Università di Parma, Parma 43124, Italy.; ^15^ Department of Integrative Biology, University of Texas at Austin, Austin, TX 78712, USA.; ^16^ Smithsonian Tropical Research Institute, Gamboa 0843-03092, Panama.

## Abstract

Divergent selection across species ranges does not always translate into observable morphological divergence. Genomic approaches agnostic to traits or groups of populations under selection can reveal cryptic genetic diversity and provide insights into the nature of local adaptation. Here, we use such an approach to investigate local adaptation in the zebra butterfly, *Heliconius charithonia*. This species is widely distributed across the Caribbean and adjacent North, Central and South America yet is morphologically and genetically homogeneous across its range. Genomic analyses, however, reveal three geographically restricted inversions that survived recent range expansions that explain genetic homogeneity in the rest of the genome. These inversions are ancient but not shared with related species. Their polymorphisms cannot be explained by accumulation of mutational load and are more likely maintained by divergent selection in heterogeneous environments. Our study emphasizes the power of genomic approaches for uncovering hidden diversity in morphologically homogeneous species and highlights the important role of chromosomal rearrangements in local adaptation.

## Teaser

Phenotype-agnostic methods detect inversion polymorphisms involved in cryptic ecological adaptation in Neotropical butterflies.

## INTRODUCTION

Understanding how species adapt to heterogeneous environments remains a central goal in evolutionary biology. Species with broad distributions are exposed to diverse selective pressures across their ranges (*1*, *2*). Such variation arises from heterogeneity of both biotic factors, including competition and predation, as well as abiotic conditions such as climate, geography, and resource availability, creating a complex and heterogeneous selective landscape that drives local adaptation (*3*). Adaptation in the face of gene flow can be achieved provided that divergent selection is strong enough to prevent loss of locally advantageous alleles and maintain a migration-selection equilibrium (*4*). Contrary to traditional views in animal evolutionary biology (*5*), gene flow has a relatively weak effect on local divergence across species ranges (*4*, *6*, *7*). Nonetheless, local adaptation can be challenging when it involves alleles at multiple loci, since gene flow can break up adaptive combinations (*8*). When adaptation is very local relative to dispersal distance, genetic architectures that reduce recombination between loci underlying adaptive traits are likely to evolve (*9*–*11*).

Structural variants (SVs), such as chromosomal inversions, are important modifiers of the recombination landscape (*8*, *12*, *13*). In heterozygotes, inversions typically suppress recombination between standard and inversion haplotypes and thereby can help maintaining linkage disequilibrium among adaptive alleles and facilitate adaptive divergence in the face of gene flow (*8*, *14*). Consistent with this, inversions and other large SVs have been increasingly implicated in a variety of adaptive phenotypes (*15*–*18*). However, much of this evidence has come from systems where conspicuous traits guided initial investigations. While such phenotype-driven studies have been invaluable for understanding the genetic basis of adaptation, they can also introduce bias in understanding the relative role of different adaptive processes and underlying genomic architecture (*19*, *20*). For instance, phenotype-first studies may inadvertently overstate the general role of large SVs in adaptation, since other SVs not linked to adaptation may remain uncharacterized. In contrast, bottom-up genomic approaches that examine genome-wide patterns of variation without relying on phenotypic assumptions offer several advantages. By being phenotype agnostic, they avoid a priori hypotheses about which groups of populations or traits are under selection, therefore providing a comprehensive and unbiased means to identify adaptive processes and their genetic underpinnings (*20*). In addition, they can be particularly helpful in organisms in which the natural history is not well studied and in species with cryptic adaptive variation (*20*).

**Fig. 1.**
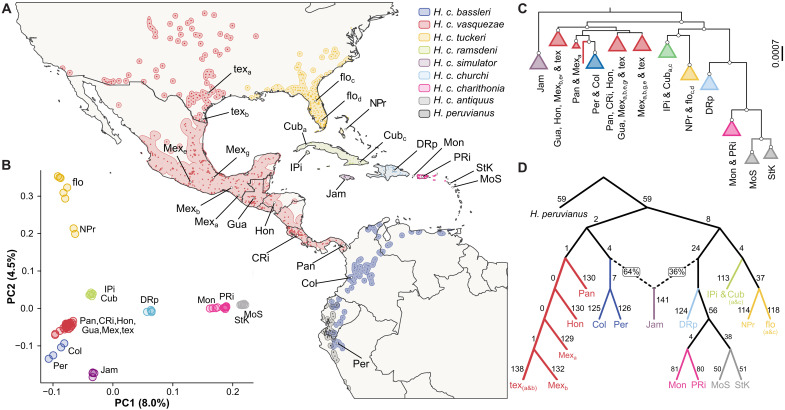
Sampling and population structure of *Heliconius charithonia*. (**A**) *H. charithonia* species distribution and sampling locations (coordinates and sampling location code names are provided in table S1). Subspecies’ ranges are depicted in different colors and were inferred on the basis of historical and current records (*136*). (**B**) PCA based on 12,394 autosomal SNPs. (**C**) ML tree based on 267,524 autosomal sites. Nodes with bootstrap support ≥70 are indicated by the white circles. Monophyletic clades of individuals from the same subspecies are collapsed. See fig. S1 for the full phylogeny. (**D**) An admixture-graph model of *H. charithonia* suggests two colonization waves of the Caribbean. Dashed edges indicate admixture events, with ancestry proportions as percentages within boxes. Solid edges and corresponding numbers indicate drift.

The zebra longwing butterfly, *Heliconius charithonia*, is widely distributed across the Caribbean and Gulf of Mexico (Fig. 1A). Its range extends from South America to southern United States, and to the Greater Antilles, and it can be found across a diverse range of habitats (rainforest edges, cloud forest, and dry forest), from sea level to ~1500 m. This species is also unique in that it is the only member of the genus found on the major Caribbean islands, which suggests dispersal abilities lacked by other *Heliconius* species. Unlike other *Heliconius*, which are iconic for the geographic diversity of wing color pattern, *H. charithonia* varies little across its extensive geographic range. Although eight subspecies are currently recognized, these are defined on the basis of very minor differences in color pattern (*21*, *22*). Genetic diversity and spatial genetic structure in *H. charithonia* remain poorly characterized, with only one previous study using low-resolution data from two mitochondrial genes, restriction fragment length polymorphisms, and allozyme data (*23*). In this study, relationships between populations were often poorly resolved because of low levels of divergence (0.4%) at mitochondrial DNA (mtDNA), suggesting a recent and rapid colonization of the Caribbean. However, given the broad distribution of this species and the breath of habitats and abiotic conditions it is exposed to, it could be a good candidate to explore cryptic adaptive processes and their genetic basis.

Here, we investigate the biogeographic history of *H. charithonia* and characterize genetic diversity across its geographic range using whole-genome sequence data. We first examine population structure and phylogenetic relationships to reconstruct the colonization history of the Caribbean. We then explore patterns of genetic variation and recombination across the genome and identify putative inversion polymorphisms. Last, we investigate the evolutionary history of these inversions and the role of selection in the maintenance of inversion polymorphisms, including potential associations between inversion haplotypes and environmental conditions across the species’ range.

## RESULTS

### Population structure and biogeographic history of *H. charithonia*

We analyzed whole-genome resequencing data from 76 *H. charithonia*, sampled from 23 locations spanning the species’ range and including representatives of all eight subspecies (Fig. 1A and table S1). Individuals group according to subspecies and geographic location, based on principal components analysis (PCA) of single-nucleotide polymorphisms (SNPs; Fig. 1B). The first two principal components explain only 12.5% of the total variation, reflecting geographic structure (Fig. 1B). Broadly, the first component distinguishes between island populations east of Cuba (Dominican Republic, Mona, Puerto Rico, Montserrat, and Saint Kitts) and all other continental and island populations, and the second component between populations north of Cuba (i.e., New Providence and Florida) from all others. The Jamaican population (*H. c. simulator*) is an outlier to other Caribbean populations and forms a separate cluster.

In a maximum-likelihood (ML) tree based on autosomal SNPs, the Jamaican population branches first from the base, suggesting that it could have resulted from an earlier colonization of the Caribbean (Fig. 1C and fig. S1A). The second deepest branch includes all the mainland populations from Peru to south Texas (*H. c. bassleri* and *H. c. vazquezae*). Relationships among the remaining crown populations suggest a stepping stone model of colonization of the Caribbean islands from mainland Central America to Cuba, thereafter following two routes: northward to New Providence and Florida and southward toward the Lesser Antilles (Fig. 1C and fig. S1A). The Z-chromosome ML tree shows broadly the same relationships as the autosomal phylogeny (fig. S1B). To further explore the possibility of multiple waves of colonization of the Caribbean, we estimated admixture graphs with varying numbers of gene flow events. In line with the hypothesis of an earlier colonization of the Caribbean, an admixture graph with a single admixture event was best supported (Fig. 1D). In this graph, the Jamaican population results from admixture between an early-diverging lineage (64% ancestry; bootstrap confidence interval: 57 to 70%)—a remnant of an older colonization event—and more recent lineages present in neighboring islands (36% ancestry, with confidence interval: 30 to 43%)—resulting from a more recent colonization. The Z chromosome retains the most variation from that earlier colonization (75.1%), compared to autosomes (25.1 to 40.6%; based on Twisst analysis; fig. S2). At the mitochondrial genome, the Jamaican population alone retains exclusively the ancestral haplotypes, the split at the base suggesting that the earlier colonization happened some 1.03 million years ago, while the extant lineages started diversifying ~620 ka ago. Diversification within the Caribbean began ~320 ka ago (based on molecular clock calibration of a Bayesian phylogenetic tree of the mitochondrial genome; fig. S3).

Levels of population differentiation and diversity are also consistent with current or recent gene flow following a recent expansion and colonization of the Caribbean islands from the continent. Population differentiation, as measured by *F_ST_*, was generally low (mean *F_ST_* = 0.096; fig. S4A) and absolute population pairwise divergence (*d_XY_* = 0.74 to 2.06%) is comparable to, albeit generally slightly higher than, within-population nucleotide diversity (π = 0.66 to 1.75%) (fig. S4B). Furthermore, levels of within-population nucleotide diversity decrease toward the edges of the distribution and are particularly low in the island populations closest to the Lesser Antilles (Mona, Puerto Rico, Montserrat, and Saint Kitts; fig. S4B). We tested the hypothesis of a recent range expansion of *H. charithonia* in two ways. First, we estimated changes in effective population size (*N*_e_) through time based both on autosomal [pairwise sequentially Markovian coalescent (PSMC)] and mitogenome [Bayesian Skyline Plot (BSP)] data. Both analyses suggest a population expansion in the recent past but at different times: ~60 ka ago (BSP; fig. S5) and 200 to 300 ka ago (PSMC; fig. S6). This is likely due to differences in the calibration of the molecular clock: The nuclear genome clock was estimated on the basis of the spontaneous mutation rate between *Heliconius melpomene* parent and offspring (*24*), while the mtDNA clock was estimated on the basis of divergence in several arthropod taxa with independently dated divergence times (*25*). Furthermore, the PSMC analysis shows that, after the initial population size increase, most populations experienced a bottleneck between ~60 and 100 ka ago, *H. c. vazquezae* populations in Central America being the exception and showing a continued increase in *N*_e_ until the recent past. We also used the directionality index (Ψ) (*26*) to infer the geographic origin and direction of the second wave of expansion. This test shows significant support for a range expansion (*P* < 0.001) with an origin in the range of *H. c. bassleri* in South America (fig. S7). This is in line with phylogenetic analysis that shows *H. c. bassleri* branches off early from the later colonization wave (Fig. 1C and fig. S1).

Together, all these lines of evidence suggest two colonization waves into the Caribbean from the mainland, with the most recent colonization wave resulting in homogenization of the genomes across the range of the species. In accord with the low levels of genetic divergence, we find a lack of hybrid sterility in crosses between *H. c. vazquezae* (Texas) with *H. c. tuckeri* (Florida)—both 32 F_1_ and 131 F_2_ hybrids, from a single pure cross and three F_1_ crosses respectively, were successfully reared until eclosion. In contrast, hybrid female sterility is often found in other interspecies and even some intraspecies crosses in *Heliconius* (*27*–*29*).

### Deeply divergent haplotypes correspond to polymorphic inversions

**Fig. 2.**
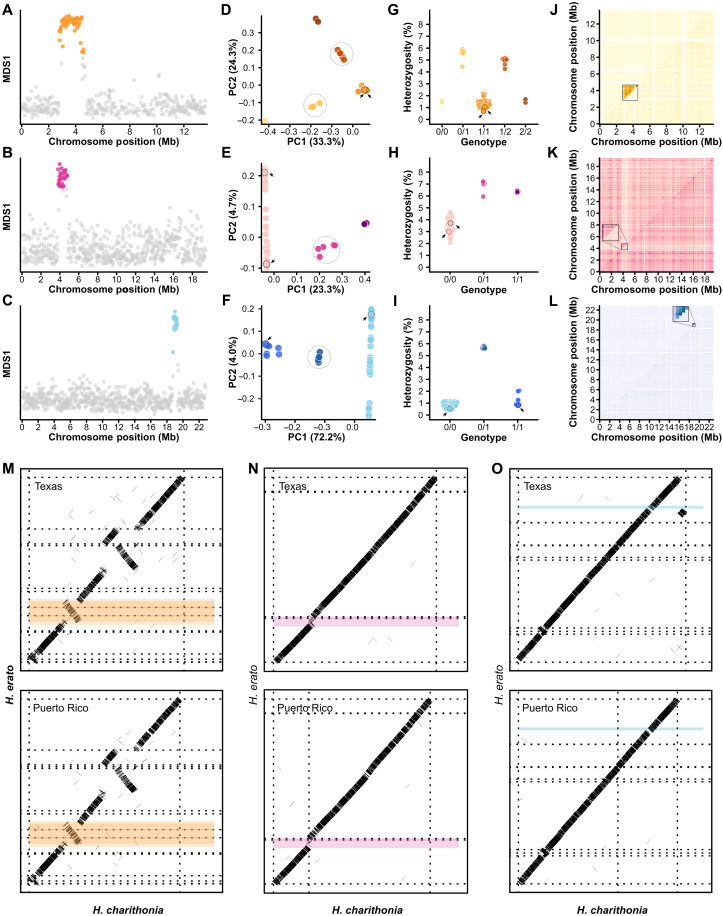
Three large nonrecombining haplotype blocks are likely inversions. (**A** to **C**) Local principal components analysis (PCA) along chromosomes 2 (A), 6 (B), and 19 (C). Each dot represents 500 SNP windows and windows with outlier multidimensional scaling (MDS) values are highlighted in orange, pink, and blue. (**D** to **F**) PCA of the outlier regions on chromosomes 2 (D), 6 (E), and 19 (F). The intermediate clusters along PC1 [(D) to (F)] and PC2 (D), highlighted by the dashed circles, likely represent individuals heterozygous for nonrecombining haplotypes. (**G** to **I**) Heterozygosity at outlier regions on chromosome 2 (G), 6 (H), and 19 (I). Each dot represents one individual. (**J** to **L**) Linkage disequilibrium for chromosomes 2 (J), 6 (K), and 19 (L). Linkage disequilibrium was calculated including all *H. charithonia* individuals (upper triangle) or only individuals homozygous for the most common homozygous genotype (lower triangle). (**M** to **O**) Alignment of the Texas *H. c. vazquezae* and Puerto Rico *H. c. charithonia* assemblies to the *Heliconius erato demophoon* reference. Only chromosome 2 (M), chromosome 6 (N), and chromosome 19 (O) are shown. Putative inversions are highlighted with different colors. Two additional rearrangements are present on chromosome 2 (M) but are not highlighted as these are known and restricted to *H. erato* (see fig. S10A). Dotted lines represent scaffold boundaries. The arrows in the PCA [(D) to (F)] and heterozygosity [(G) to (I)] plots point to the *H. charithonia* reference genome individuals. Texas, circled with solid black; Puerto Rico, dashed black circle.

The lack of strong overall population structure in *H. charithonia* allows the detection of outlier genomic regions that may be under selection. We used a local PCA approach along the genome to identify genomic regions with distinct population structure. This approach has the advantage of not requiring any a priori definition of which population or groups of populations might be differentiated. Using this method, we found three large outlier genomic regions: one on chromosome 2 (~1.85 Mb), another on chromosome 6 (~1.00 Mb), and a third on chromosome 19 (~540 kb; Fig. 2, A to C, and fig. S8).

On chromosome 2, the PCA of the outlier region shows five distinct clusters (Fig. 2D). The two clusters occupying intermediate positions along PC1 and PC2 space (dotted circles) have the highest heterozygosity (Fig. 2G). Both the intermediate clusters and the two peripheral clusters with the least common genotypes (leftmost of PC1 and top of PC2) are found only in *H. c. vazquezae* (from Panama to Texas); the other peripheral cluster (right of PC1) includes both *H. c. vazquezae* and all individuals from the other subspecies (table S1). These findings are consistent with the existence of three groups of individuals homozygous for distinct nonrecombining haplotypes, the two intermediate clusters representing heterozygotes and with another group being absent from our dataset. The chromosome 6 and 19 outlier regions yield three very distinct clusters (Fig. 2, E and F), the intermediate clusters along PC1 (dotted circles) having highest heterozygosity in both cases (Fig. 2, H and I). The peripheral clusters likely represent homozygotes for alternative haplotypes. Both the intermediate and the peripheral cluster with the least common genotypes include only *H. c. vazquezae* individuals (table S1). In all three cases, linkage disequilibrium is high across these outlier regions when analyzing all individuals together but not when analyzing only individuals from the most abundant cluster (Fig. 2, J to L). Genetic divergence (*d_XY_*) between individuals of the three peripheral clusters on chromosome 2 and the two peripheral clusters on chromosome 6 and 19 (i.e., homozygotes) is ~5.30 to 6.05, 5.47, and 6.51% within these regions, far exceeding divergence within clusters (1.87 to 2.43%, 2.81 to 4.28%, and 1.59 to 2.05% for chromosome 2, 6, and 19, respectively; fig. S9). These patterns are consistent with large, divergent structural rearrangement polymorphisms that suppress recombination between haplotypes, allowing accumulation of divergence between them while maintaining linkage disequilibrium along the haplotype.

To determine whether these divergent haplotypes are associated with SVs, we compared two genome assemblies of *H. charithonia* (from Texas and Puerto Rico) to other *Heliconius* chromosome (or near-chromosome)–level genome assemblies. Both individuals share the same genotype at the outlier regions on chromosome 2 and 6, being homozygous for the most frequent haplotype in *H. charithonia* (Fig. 2, C and D). Genome alignments show that both individuals are homozygous for an inversion at chromosome 2, which overlaps the haploblock region, with breakpoints at positions at Herato0206:503,660 and Herato0209:708,130 (Fig. 2M and fig. S10A). Hence, the most frequent haplotype in *H. charithonia* represents the inverted state of the region. We further examined the breakpoints of the haplotypes underlying the five clusters in the outlier region of chromosome 2 by visualizing genotypes across this interval (fig. S11A). The largest haplotype corresponds to the inversion identified in the genome alignments and predicted by the local PCA. A second haplotype, spanning ~1.46 Mb, is fully nested within the larger inversion. This structure likely reflects a secondary inversion, restoring the orientation of the nested region to the ancestral state (fig. S11B). Recombination in heterokaryotypes anywhere within this inversion region will likely generate unbalanced gametes, as gametes would carry both duplications and deletions (see fig. S11C for details). Furthermore, the high levels of divergence all along the inversion region suggests that recombination has been strongly suppressed.

At the outlier region on chromosome 6, both *H. charithonia* assemblies share the same genotype (homozygous for the most frequent haplotype), but neither shows evidence of an inversion based on genome alignments (Fig. 2N and fig. S10B). While this could indicate that an inversion is instead associated with the least frequent haplotype (an assumption we adopt hereafter), the absence of signal could also result from genome alignment in this region being particularly poor (Fig. 2N). Elevated nucleotide diversity (π) within both clusters of homozygotes could also suggest a more complex rearrangement (e.g., duplications) within the region (fig. SS9).

Last, at the outlier region on chromosome 19, the *H. charithonia* assemblies are homozygous for different genotypes (Fig. 2F), yet we found no evidence of an inversion based on genome alignments to other *Heliconius* genomes (Fig. 2O and fig. S10C). However, this is likely due to a difficulty in aligning within this region due to repetitive content (fig. S12) and/or misassemblies in and around putative inversion breakpoints (Fig. 2O and fig. S10C). Illumina short-read coverage is elevated near the boundaries of the chromosome 19 region relative to the genome-wide average, suggesting that the breakpoints are rich in repetitive sequences (fig. S12). Consistently, both *H. charithonia* genome assemblies align poorly to a ~180-kb region (Herato1910:1,521,759-1,701,968) within the putative inversion (Herato1910:1,494,502-2,034,941) in the *Heliconius erato* reference genome (Fig. 2O). Genome assembly across repetitive regions can be challenging, particularly when generated from short-read data, as in the Puerto Rico assembly (which used 10X Chromium technology). The Texas assembly was generated using PacBio and Hi-C data, which should allow correct assembly provided that the reads were long enough to span the repetitive regions around inversion breakpoints and/or Hi-C data maps uniquely to both sides of the inversion breakpoints (*30*). However, mapping of Texas Hi-C data around the left breakpoint of the putative inversion regions is poor (both when mapped to its own assembly or the Puerto Rico assembly), yielding no detectable signal of an inversion (fig. S13). While we cannot directly show that the haploblocks on chromosome 6 and 19 are inversions and/or could correspond to a more complex or a series of chromosomal rearrangements, for simplicity, we will refer to all these regions as inversions.

### Evolutionary history of the inversions

**Fig. 3.**
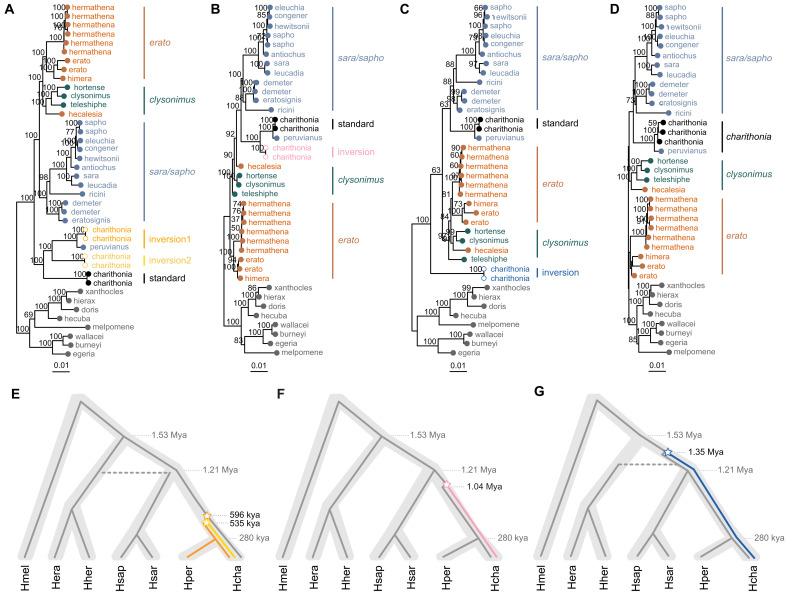
Phylogenetics of polymorphic inversions and collinear regions of the genome. (**A** to **D**) ML phylogenies of inversion regions on chromosome 2 (A), chromosome 6 (B), chromosome 19 (C), and whole-genome collinear regions (D). Trees were rooted using midpoint rooting. Bootstrap values indicated next to nodes. Outgroup individuals are colored according to the main clades: *erato* (red), *sara*-*sapho* (blue), *clysonymus* (green), and *melpomene/doris/burneyi* (gray). *H. charithonia* individuals are colored according to their inversion haplotypes: inversion (gold, pink, and dark blue) and standard (black). (**E** to **G**) Inferred evolutionary scenarios for the inversion haplotypes on chromosomes 2 (E), 6 (F), and 19 (G). The likely origin of inversion haplotypes (indicated by stars) and their estimated age (indicated in black) are shown against the species tree inferred from collinear regions of the genome (gray background; relevant node ages are indicated in gray). Node ages were estimated using a mutispecies coalescent approach (see table S2). Dashed lines in the genealogy of inversion regions [(E) and (G)] indicate probable introgression that explains the differences from the main species tree (D).

To investigate the origins of the divergent haplotypes at the three inversions, we compare with closely related species from the *erato*, *clysonymus*, and *sara*/*sapho* clades (table S1). Deep divergence between the *H. charithonia* haplotypes at the three inversions compared to genomic background levels of divergence (fig. S9) could be explained by introgression from other species. To test the latter, we calculated *d_XY_* along chromosomes between *H. charithonia* homozygotes and individuals representative of outgroup species. We found no drop in *d_XY_* at any of the inversion regions as would be predicted by recent introgression (figs. S14 to S16). Furthermore, ML trees show that none of the inversion haplotypes group with any other species (Fig. 3). On chromosome 2, we find both standard and the two inversion haplotypes are basal to the whole *erato*-*clysonymus*-*sara/sapho* clade (Fig. 3A). Also, all three haplotypes are deeply divergent, but the largest *H. charithonia* inversion haplotype (primary inversion), which groups with *Heliconius peruvianus*, is more closely related to the nested (secondary) inversion than to the standard arrangement. This topology indicates that both inversion haplotypes are derived relative to the standard arrangement. On chromosome 6, both standard and inversion haplotypes group together and branch deep in the *sara/sapho* clade (Fig. 3B), as in the majority tree inferred from collinear regions of the genome (Fig. 3D). On chromosome 19, one haplotype—presumed to carry the inversion—forms the deepest branch in the whole *erato* + *clysonymus* + *sara/sapho* clade (Fig. 3C). In contrast, the branch of the alternative haplotype—presumed to represent the standard arrangement—retains its position deep in the *sara*/*sapho* clade as seen in the average autosomal tree (Fig. 3C). The same results were also obtained when using a coalescent-aware method to estimate species trees in blocks along chromosomes (fig. S17).

An alternative explanation for the elevated divergence at these inversions is an ancient origin of the inversion haplotypes (i.e., retention of an ancestral polymorphism). In particular, the phylogenies of the inversion regions on chromosomes 2 and 19 (Fig. 3, A and C), which are inconsistent with the tree inferred from collinear regions of the genome (Fig. 3D), and where inversion haplotypes branch most deeply in the entire *erato*-*clysonymus*-*sara*/*sapho* clade, would suggest that they predate the diversification of the group. To investigate the timing of origin of the inversions, we estimated the ages of the inversion polymorphisms and compared these to divergence times estimated under the majority tree from collinear regions (Fig. 3D and table S2). In all three cases, their origin predates the split between *H. charithonia* and its sister species *H. peruvianus* (~300 ka ago), supporting an ancient origin, but still more recent than the root age of *erato* + *clysonymus* + *sara/sapho* clade of 1.53 Ma ago estimated from collinear parts of the genome. The inversions on chromosome 19 and 6 originated 1.35 and 1.04 Ma ago, respectively, while both primary and secondary inversion haplotypes on chromosome 2 had a more recent origin at 596 and 535 ka ago.

The conflicting patterns between the species tree and inversion topologies (and dates) on chromosomes 2 and 19 can instead be reconciled if we assume introgression between clades not carrying the inversion haplotypes (Fig. 3, E and G). In the case of the chromosome 2 inversion, introgression from an ancestor of the *erato* (or *clysonimus*) clade into an ancestor of the *sara/sapho* clade, after the latter split from *H. peruvianus* and *H. charithonia* (Fig. 3E), would render both clades (*erato* and *sara/sapho*) sister to each other rather than the latter being sister to *charithonia*/*peruvianus* as in the rest of the genome (Fig. 3D and fig. S17). Regarding the putative chromosome 19 inversion, the posterior mean estimate for its origin (1.35 Ma ago) is slightly younger than the root age of the *erato-clysonymus-sara/sapho* clade (1.53 Ma ago), though their confidence intervals overlap (table S2). However, node ages estimated from collinear parts of the genome (including root age) could be underestimated since our analysis does not account for gene flow, which is common in this clade (*31*). Moreover, since the arrangements are likely under divergent selection, their age could be overestimated. Therefore, while it remains possible that the chromosome 19 inversion predates the origin of the entire group, it is more likely it originated after the split between the *erato-clysonymus* and *sara/sapho-charithonia* clades. The discordant topologies can then be reconciled assuming introgression between the common ancestor of *sara*/*sapho*-*charithonia* clades, not carrying the inversion, and the common ancestor of the *erato-clysonymus* clade (Fig. 3F).

In sum, all three inversion polymorphisms appear to be old. Given their ages and that none is shared with other species, the inversions appear to have been maintained as long-term polymorphisms as a result of some sort of balancing selection since their origins deep in the ancestry of *H. charithonia* lineage.

### Little evidence for deleterious effects of inversions

To explore possible deleterious effects of the inversions, we first investigated the inversion breakpoints (table S3). Inversions can disrupt genes if breakpoints fall within a gene or its regulatory elements (*32*). Of the two inversion breakpoints of the largest (primary) inversion on chromosome 2, inferred on the basis of genome alignments, only the right breakpoint falls within a gene (evm.TU.Herato0209.48), an ortholog of CG31344 in *Drosophila melanogaster*. For the nested (secondary) inversion on this chromosome, both inversion breakpoints (inferred on the basis of local PCA and visualization of genotypes) fall within genes (evm.TU.Herato0206.24 and evm.TU.Herato0209.31, for the left and right inversion breakpoints, respectively). Their orthologs in *D. melanogaster* (CG31229 and *Arc42*, respectively) are both associated with mitochondrial function. On chromosome 6 and 19, we obtained only approximate coordinates of the inversion breakpoints based on the local PCA. The breakpoints on chromosome 6 do not overlap annotated genes, and the closest genes are 106 and 130 kb from left and right breakpoints. On chromosome 19, the first breakpoint is within the gene evm.TU.Herato1910.104, an ortholog of *PIH1 domain containing 1* in *D. melanogaster*. The second inversion breakpoint is within evm.Herato1910.121 orthologous or paralogous to *highroad* (*hiro*) and *CG32483* genes in *D. melanogaster*.

**Fig. 4.**
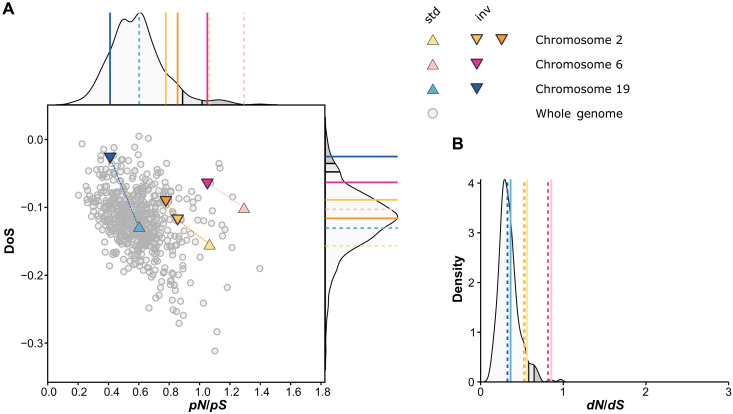
Limited evidence for accumulation of deleterious mutations in inversions. (**A**) DoS and ratio of nonsynonymous to synonymous polymorphisms (*pN*/*pS*), computed in 500-kb windows genome-wide and in the inversion regions. (**B**) Ratios of nonsynonymous to synonymous substitutions (*dN*/*dS*). The ancestral and derived state of the inversions are given by the dashed and full colored lines. Shades of gray are used to display 0.95 and 0.975 quantiles of the genome-wide values. std, standard; inv, inversion.

We next investigated possible deleterious mutational load carried by the inversions, which can accumulate because of reduced efficacy of purifying selection resulting from suppressed recombination in heterozygotes (provided that these are common). We estimated the rate of nonsynonymous to synonymous polymorphism (*pN*/*pS*), the rate of nonsynonymous to synonymous substitution (*dN*/*dS*), and the directionality of selection (DoS), for both inversion and standard haplotypes independently. Inversion haplotypes present levels of nonsynonymous relative to synonymous polymorphism (*pN*/*pS*) and substitution (*dN*/*dS*) in line with the whole genome (Fig. 4, A and B). While levels of nonsynonymous over synonymous substitution (*dN*/*dS*) are similar to those of standard haplotypes (Fig. 4B), the ratio of nonsynonymous to synonymous polymorphisms (*pN*/*pS*) is lower in inversion haplotypes (Fig. 4A). Correspondingly, inversion haplotypes show overall negative (i.e., purifying) selection (DoS_chr2_1_ = −0.09, DoS_chr2_2_ = −0.12, DoS_chr6_ = −0.06, and DoS_chr19_ = −0.03), although less negative than the standard haplotypes (Fig. 4A). This pattern is consistent whether the inversion haplotype is the most frequent allele (primary inversion on chromosome 2) or least frequent allele (secondary inversion on chromosome 2 and inversion haplotypes on chromosome 6 and 19). These results suggest that the inversions do not harbor a strong deleterious mutational load and some nonsynonymous mutations in the inversion haplotypes could be positively selected.

The lack of signal of accumulation of strongly deleterious variants could be explained by rare recombination and/or gene conversion in heterokaryotypes (i.e., gene flux). Gene flux decreases linkage-disequilibrium within inversions, helping mitigate some of the costs associated with recombination suppression (*33*–*35*). To explore this hypothesis, we attempted to quantify gene flux between the inversion haplotypes by fitting demographic models of strict divergence and isolation with migration using gIMble. In all three inversions and all inversion haplotype pairwise comparisons, a model of strict divergence is favored (table S4). To examine potential gene flux at a finer scale, we also performed topology weighting (Twisst) analysis in sliding windows along the inversions. In all three inversions, topologies grouping individuals by haplotype (rather than geographic proximity as expected because of gene flux) were strongly supported (weight ≥ 0.95) (figs. S18 to S20).

If inversion haplotypes accumulate recessive mutational load, inversion homozygotes should be rare because of heterozygous advantage. Instead, heterokaryotypes seem rare in all three inversions (*n* = 15, 5, and 6, of 76 individuals in chromosomes 2, 6, and 19, respectively) (table S1). Focusing only on *H. c. vazquezae* from Central America and Texas, the only subspecies in which the inversions are polymorphic, inversion genotype frequencies deviate significantly (*P* < 0.01) from Hardy-Weinberg equilibrium (HWE) in all inversions because of a deficit of heterokaryotypes. The only exception is one class of heterokaryotypes on chromosome 2, which are more frequent than expected (table S1). Because recombination proceeds normally in inversion homozygotes, deleterious mutations can be efficiently purged once an inversion reaches moderate to high allele frequencies, providing a plausible explanation for the absence of signals of mutational load seen in this system. Overall, evidence thus suggests that none of the inversions is associated with strongly deleterious fitness effects. The lack of heterozygotes is instead consistent with local selection favoring homozygotes, although our ability to test this hypothesis was limited by the small number of samples available per population.

### Inversions maintained by spatially heterogeneous selection

**Fig. 5.**
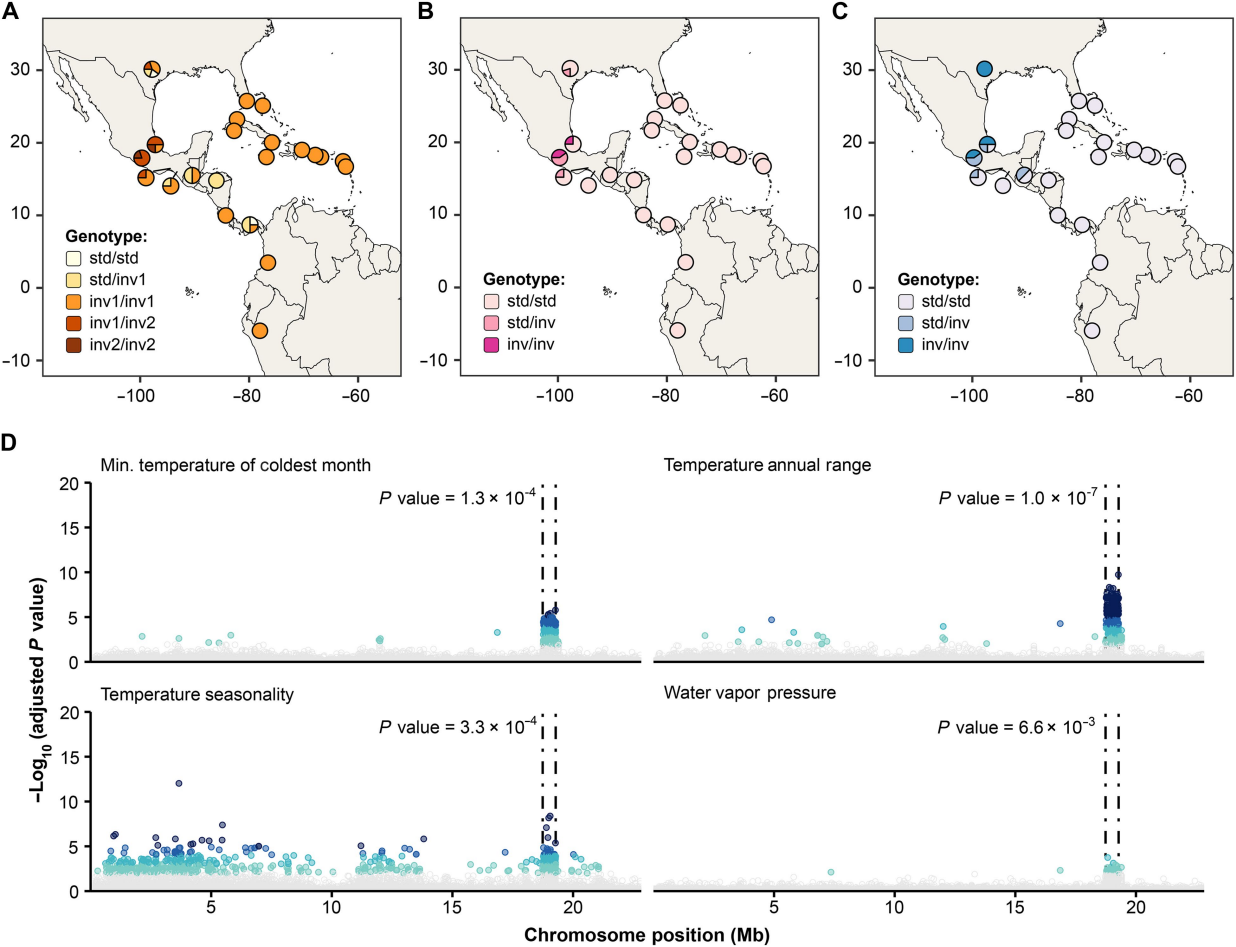
Association of inversion haplotypes with environmental factors. (**A** to **C**) Geographic distribution of chromosome 2 (A), chromosome 6 (B), and chromosome 19 (C) inversion genotypes. (**D**) Latent factor mixed-models Manhattan plots showing association with climatic variables inside chromosome 19 inversion. Each point indicates *P* values at each SNP. Points are colored according to estimated false discovery rates (dark blue, <0.00001; medium dark blue, <0.0001; light blue, <0.001; gray, ≥ 0.001). *P* values for the inversion coded as a single-locus are also shown. The dashed vertical lines represent the inferred boundaries of the inversion.

Inversion polymorphisms may persist over the long term in a heterogeneous environment if local selection favors distinct haplotypes in different regions. The three inversions are polymorphic only in *H. c. vazquezae*. On chromosome 2, the rare haplotypes are found only in part of the range of this subspecies: The ancestral haplotype is mostly found in the southernmost *H. c. vazquezae* populations in our dataset, from Panama to Chiapas near the southern border of Mexico (with one occurrence in Texas), while the secondary inversion is found only further north near the mountainous regions, mostly from Oaxaca and Veracruz in Mexico to Texas (Fig. 5A). The inversion on chromosome 6 also has a restricted distribution, being mostly restricted to Oaxaca in Mexico to Texas (Fig. 5B). The inversion on chromosome 19 seems to follow a south-north gradient, with one copy present in Guatemala and southern Mexico each, and becoming almost fixed further north in Mexico (Oaxaca and Veracruz) and fixed in our Texas sample (Fig. 5C).

The geographically restricted distributions of these rare inversions are consistent with local adaptation, particularly the chromosome 19 inversion. SNPs within the chromosome 19 inversion are significantly associated with four environmental variables: minimum temperature of the coldest month, temperature seasonality, temperature annual range, and water vapor pressure (Fig. 5D and fig. S21). Individuals homozygous for the inversion on chromosome 19 face significantly lower temperatures in the coldest month (3.9° to 10.8°C) and greater temperature oscillations throughout the year (temperature seasonality = 165 to 708; temperature annual range = 19° to 31°C), compared with homozygotes for the ancestral arrangement (minimum temperature in the coldest month = 8.0° to 22.8°C; temperature seasonality = 26 to 518; temperature annual range = 8° to 25°C; fig. S22 and table S5). Water vapor pressure, a proxy for humidity, is also significantly lower in regions where inversion homozygotes are found (1.4 to 1.9 kPa) than in other geographic locations (1.6 to 2.9 kPa). The rare, inversion haplotype on chromosome 19, present mainly in the northern part of the distribution, seems likely to confer a selective advantage for particularly cold and dry conditions in subtropical Texas and Central and Northern Mexico. SNPs within the chromosome 2 and 6 inversions, in contrast, show no obvious association with climatic variables (figs. S21, S23, and S24).

## DISCUSSION

Here, we present a genome-wide analysis of *H. charithonia* population structure, with a focus on its biogeographic history and geographic distribution of genetic diversity. Using resequence data sampled across the species range, we infer two colonization waves of the Caribbean from the mainland into the Greater Antilles. The most recent colonization resulted in homogenization of genetic diversity across the whole species range, consistent with the low morphological diversity of this species. Using unsupervised methods to detect regions of the genome with outlier genetic structure, we found ancient large inversions segregating in *H. charithonia*, one of which likely plays a role in local adaptation to climatic conditions at the subtropical edge of its range.

### Genetic homogeneity and colonization of Caribbean islands

Unlike other *Heliconius* species with wide distributions, population structure is weak in *H. charithonia*. For example, considerable population structure exists across broad biogeographic scales in *H. erato* and *H. melpomene* (*36*, *37*). Deep genetic divergence in those species is often associated with major geographic barriers, for example between populations across the Andes. Within biogeographic regions, there is little differentiation across most of the genome, even between geographic populations with different wing color patterns (*38*, *39*). In *H. charithonia*, these major barriers do not seem to disrupt genetic homogeneity, nor do we find major ecological differences between mainland and island habitats. *H. charithonia* exhibits very little local population structure (*40*) and regularly disperses hundreds of kilometers north within a single year from south of the Rio Grande valley in Northern Mexico to Central Texas and beyond (*41*) (Fig. 1A). This long-range dispersal ability likely explains why the species is so genetically homogeneous, at least on the mainland. Relatively recent, rapid expansion into the Caribbean from the continent would explain the low levels of genetic divergence there as well. We identified at least two colonization waves of the Caribbean by *H. charithonia* from our genomic dataset. The most recent colonization of the Caribbean (~180 to 320 ka ago) likely occurred from Central America into Cuba, through the Yucatán Peninsula. From there, *H. charithonia* expansion split into two routes: north into Florida and the Bahamas and south into the Greater Antilles (Fig. 1D).

An earlier colonization (~1.03 Ma ago) can also be inferred from genetic variation found exclusively in Jamaica. This is consistent with another study on birds showing that this island is more isolated than other Antillean islands, retaining variation from earlier Caribbean colonizations (*42*). Evidence of an ancestral colonization comes mainly from mitochondrial genomes (all four Jamaican individuals show similar deeply divergent haplotypes) and from genetic variation at the Z chromosome (75.1% ancestral variation). In contrast, the autosomes retained much less ancestral variation (31.4%). The contrast between autosomal and mitochondrial loci was suggested to result from male-biased migration into the Caribbean and/or different effects of founder events and genetic drift on the different genomic compartments (*23*). Our data from the Z chromosome allows us to test between these hypotheses. In ZW systems such as *H. charithonia*, male-biased dispersal would favor an excess—not a deficit—of homogenization of variation on the Z chromosome (males are ZZ). Instead, the ancestral variation in different genomic compartments is more consistent with differential impact of founder events and genetic drift and/or the Z carrying more incompatibilities than autosomes (*43*).

### Evidence for chromosomal inversions

The availability of population-level whole-genome datasets allows the discovery of SVs in a comprehensive and cost-effective way (*20*). For example, an increasing number of studies have investigated genetic heterogeneity along the genome using unsupervised methods such as local PCA in sliding windows (*44*). Such methods have revealed genomic blocks of tightly linked SNPs that are highly differentiated, providing clues to SVs such as chromosomal inversions that reduce recombination (*45*–*50*). Complementary analyses, including PCA within these regions showing discrete genetic clusters and elevated heterozygosity of the putative heterokaryotypes, further support the presence of an inversion polymorphism. Our unsupervised approach revealed three highly differentiated genomic regions that are very likely inversions. Two nested inversions, the larger 1.9 Mb long and the smaller 1.5 Mb, fall on chromosome 2. Inversions on chromosome 2 have been described previously in other *H. erato–clysonymus–sara/sapho* species (*50*–*53*), but the inversion in *H. charithonia* is not homologous to any of these. The putative inversions on chromosomes 6 and 19 are ~1 Mb and 540 kb long, respectively, and have not been previously described in *Heliconius*.

Uncovering inversions from indirect evidence has drawbacks. Since indirect methods rely on signals of linkage and divergence, they most easily detect large, polymorphic, and highly divergent inversions (*20*). It is thus likely that our dataset does not fully describe all structural variation present in *H. charithonia*. In addition, inversions are best confirmed using direct methods such as long-read sequencing and/or Hi-C sequencing data (*54*, *55*). Direct methods are also better for characterizing inversion breakpoints, which can only be approximated using indirect approaches. We directly confirmed the largest inversion on chromosome 2 and determined its breakpoints (Fig. 2M) but could not do so for the smaller nested inversion on chromosome 2 or the other putative inversions on chromosomes 6 and 19 (Fig. 2, M to O). Our inability to confirm the chromosome 6 inversion may be explained if neither *H. charithonia* assembly (from Puerto Rico and Texas) carries the inversion haplotype (both are homozygous for the same haplotype; Fig. 2E). Also, all three genotypes show particularly high diversity within this region when compared with the genome background (Fig. 2H and fig. S9B), which could indicate further hidden structural complexity not captured by our approaches. In contrast to the two other outlier regions, the two *H. charithonia* assemblies have distinct genotypes at the chromosome 19 region (both homozygous, but with different inversion haplotypes), yet genome alignments show them to be collinear with outgroup species. While it is possible that this region does not correspond to an inversion, we believe that we were unable to confirm it because of a misassembly in one of the reference genomes, most likely the Texas assembly (i.e., the inversion was not assembled in the correct orientation) due to the complex genomic structure around the putative inversion region (Fig. 2O and figs. S12, S13, and S25). Furthermore, and consistent with assembly problems near this breakpoint, a ~500-kb region directly adjacent and upstream of the putative inversion is duplicated in the Texas assembly and is also present at the end of chromosome 19 (figs. S9 and S25). When mapping PacBio reads to its own genome assembly, coverage in these regions drops to half the genome-wide coverage (fig. S25), suggesting that they do not represent a true duplication/translocation but instead two distinct haplotypes of the same genomic region. Because of high divergence, they were assembled separately, with one being placed at the end of chromosome 19 (fig. S25). This underscores the complex genomic structure around the putative inversion region, and we cannot rule out the possibility of misassembly. Overall, our results provide strong evidence for multiple SVs in *H. charithonia*, most likely inversions, although the difficulty in confirming several candidates suggests that the genomic architecture of some of these regions may be more complex.

### Ancient polymorphic inversions involved in adaptation to heterogeneous environments

Chromosomal inversion polymorphisms are often ancient and may predate the origin of the species in which they are found (*12*). This is sometimes explained by introgression from closely related species (*56*–*59*), including in *Heliconius* (*50*–*52*, *60*). We find that inversions segregating in *H. charithonia* are old (535 ka ago to 1.35 Ma ago), all three predating the split between *H. charithonia* and its sister species *H. peruvianus.* However, we failed to find any evidence of introgression and it seems more likely these inversions are ancient ancestral polymorphisms.

The long-term persistence of inversion polymorphisms raises questions about how inversions become established and maintained within species. Multiple factors—including local adaptation, mutation load, and breakpoint effects—have been suggested to explain these processes, and their relative importance may change through the life of an inversion (*8*, *14*, *32*). For instance, in *Heliconius numata*, wing-color polymorphism is associated with an inversion at a mimicry locus (supergene *P*) on chromosome 15 (*15*, *61*). In this system, Müllerian mimicry adaptations associated with recombination suppression are thought to explain the initial spread of the inversion polymorphisms, but this may be coupled with assortative mating among phenotypes and accumulation of deleterious mutations leading to heterozygote advantage (*62*). In *H. charithonia*, there is little evidence for accumulation of mutational load in the form of nonsynonymous mutations in any of the inversions. In the absence of recombination, deleterious mutations are expected to accumulate in inversions that are frequently heterozygous, and each arrangement may fix different mutations, leading to greater fitness of heterozygotes. Recombination is suppressed only in heterokaryotypes but can proceed in homokaryotypes, so that purifying selection can remove deleterious mutations when both chromosomal morphs maintain reasonably large effective population sizes (*63*). In *H. charithonia*, heterokaryotypes were rare in all inversions (*n* = 15, 5, and 6, on chromosomes 2, 6, and 19, respectively) across the species (*n* = 76) (Fig. 5 and table S1). Even within *H. c. vazquezae*, the only subspecies in which all three inversions are polymorphic, inversion genotypes show no evidence of heterozygote excess, suggesting that neither inversion haplotype has fixed highly deleterious mutations. Demographic modeling and phylogenetic analyses indicate that the absence of mutational load cannot be explained by gene flux between haplotypes (table S4 and figs. S18 to S20).

A more plausible explanation for the establishment and maintenance of inversion polymorphisms in *H. charithonia* is local adaptation. Inversions can be maintained as stable polymorphisms in a heterogeneous geographical landscape by migration-selection balance (*8*, *64*). If a new inversion captures a set of locally adapted alleles at two or more loci, the inversion haplotype will be advantageous, since suppressed recombination within the inversion preserves the favorable combination of alleles (*8*). The spatial structure of the chromosome 19 polymorphism and its association with environmental conditions (Fig. 5, A and C) suggest that different inversion haplotypes confer local benefits in response to climatic conditions. Weak geographic structure at the whole-genome level indicates that populations are currently connected by high levels of gene flow, which, in the inversion region, is counteracted by divergent selection. Notably, the chromosome 19 inversion haplotype present in Mexico and Texas resisted a recent population expansion that homogenized most genetic variation across the entire species range. While it is unclear whether this inversion was initially advantageous and spread because of recombination suppression, it is likely currently maintained by a balance between migration and selection. Clinal variation of inversions across geographical or environmental gradients offers compelling evidence of natural selection driven by abiotic factors (*12*, *65*). Numerous recent examples of species in which chromosomal inversions segregate between distinct ecotypes or in parallel with environmental gradients include monkeyflowers (*66*), sunflowers (*45*, *46*), deer mice (*47*), seaweed flies (*48*), annual ragweeds (*49*), marine snails (*18*, *67*), cod (*34*), sticklebacks (*68*), and other *Heliconius* butterflies (*50*, *53*). Likewise, in *H. charithonia*, the three inversion polymorphisms exhibit geographic structuring, suggesting a potential role in local adaptation.

Several lines of evidence indicate that the chromosome 19 inversion confers a local adaptive advantage, particularly in response to cold and desiccation stress. First, genotype-environment analyses show an association between SNPs within the inversion and climatic variables related to temperature variability, cold, and desiccation (Fig. 5D and fig. S21). A previous study also found that temperature was a key factor determining residency time of *H. charithonia* in Texas during warmer months (*41*). Second, the inversion polymorphism appears to follow a south-north gradient and is spatially segregated between populations in dry, cold habitats (restricted to Texas and Mexico) and populations in warmer, more humid habitats (elsewhere in the distribution; Fig. 5C and fig. S22). Third, the chromosome 19 inversion contains 18 genes (table S3), including two near inversion boundaries—*Catalase* (*Cat*) and *Trehalose transporter 1*-*like* (*Tret1l*)—that have been implicated in adaptation to cold and desiccation across different taxa (*69*–*73*). In insects, species resistant to cold also tend to be tolerant of desiccation (*74*, *75*), and many mechanisms underlying tolerance to these two environmental stresses overlap (*74*). These include up-regulation of antioxidant defenses and metabolism and transport of trehalose between cells and the hemolymph. Periods of environmental stress, such as cold and desiccation, result in an increase of reactive oxygen species that can cause cellular damage (*75*, *76*). In response, increased activity or expression of antioxidant enzymes helps mitigate oxidative stress, including catalase that breaks down harmful hydrogen peroxide into water and oxygen (*69*, *70*, *77*). Another key response to extreme conditions, such as cold, heat, and desiccation, is biosynthesis and transport of sugars, especially trehalose. Trehalose, the main hemolymph sugar in most insects, acts as a cryoprotectant at low temperatures. Its accumulation improves tolerance to cold, desiccation, and hypoxia and facilitates cryoprotective dehydration by replacing water and preserving the structure of proteins and membranes during stress (*78*–*81*). Given its geographic structure, association with environmental variables, and functional gene content, the chromosome 19 inversion polymorphism is highly likely involved in adaptation to drier, colder subtropical conditions in northern populations of *H. c. vazquezae*, enabling its success as the most northerly distributed member of its genus.

The inversion polymorphisms on chromosome 2 and 6 showed no association with any of the climatic variables that we examined. The outlier region on chromosome 6 encompasses multiple gene copies orthologous to the *timeout* gene in *D. melanogaster*. The paralog of this gene in insects, *timeless*, is a canonical circadian clock gene (*82*, *83*). The role of *timeout* as a circadian clock gene is more contentious, although it has been reported to be involved in light entrainment of the circadian clock in *Drosophila* (*83*), variation in circadian activity rhythms in different populations of rice borer moths (*84*), and diapause thresholds and voltinism in speckled wood butterfly (*85*). *Heliconiini* butterfly adults, including *H. charithonia*, frequently exhibit promenading behavior, but their activity seems to depend on external cues (e.g., light levels and/or temperature) that could also be related with periods when predators are most active (*86*–*88*). It has also been shown that *Heliconius* can use time as a context for making foraging decisions related with availability of pollen resources that vary predictably in time in different host plants (*89*). It is thus possible that the polymorphism at the chromosome 6 inversion could be linked to changes in behavior in response to external cues and/or resource availability. Regarding the large inversion on chromosome 2, a Gene Ontology enrichment analysis revealed an overrepresentation of genes associated with “wing disc” and “gustatory receptor neuron” phenotype categories. The latter phenotype raises the intriguing possibility that the chromosome 2 inversion may play a role in host plant adaptation. Notably, *H. charithonia* is among the few *Heliconiini* adapted to and able to feed on *Passiflora* hosts with hooked trichomes (*90*, *91*). Larvae from mainland populations (*H. c. vazquezae*) can escape physical damage from the trichomes and feed on the host plant because of external (thicker and more puncture-resistant cuticle) and postingestive adaptations (*90*–*92*). Coincidently, the distribution of *Passiflora* species with hooked trichomes, such as *Passiflora adenopoda* and *Passiflora lobata*, is restricted to northern South America and Central America. These *Passiflora* overlap with the range of *H. c. vazquezae* (Central America), where the chromosome 2 inversion haplotypes are common but also extends to that of *H. c. bassleri* (South America), which is fixed for the same inversion haplotype found on the island populations. Several factors may explain the lack of perfect association between the ranges of inversions haplotypes and trichome-bearing *Passiflora*: (i) Variation in relative densities of *Passiflora* with and without trichomes (which exist in these areas) may change through Central and South America; (ii) we might have missed the inversion in *H. c. bassleri* because of low sample size (*n* = 4); or (iii) the chromosome 2 inversion is not involved in host adaptation. Experimental assays and genetic mapping will be fundamental to directly test links between inversion haplotypes and putative adaptive traits, as demonstrated in other species (*17*, *48*, *93*). This should be particularly feasible in this system, given the low genomic divergence outside inversion regions and full fertility observed between subspecies.

## MATERIALS AND METHODS

### Sample collection and genome resequencing

We performed Illumina short-read whole-genome sequencing of 76 *H. charithonia* collected from across most of *H. charithonia* native range (Fig. 1A and table S1). These included wild-caught specimens collected from different localities across the species ranges between 1990 and 1991; wild-caught samples collected in Peru (2011), Honduras (2022), Guatemala (2023), and Mexico (2016 to 2019, 2021, and 2023); and fresh samples reared in captivity from Colombia, Costa Rica, and Texas (table S1). RNA-free genomic DNA was extracted using the E.Z.N.A Tissue DNA kit (Omega Bio-tek Inc.), including a ribonuclease A treatment step. Samples from Honduras, Guatemala, and Mexico were extracted using a magnetic bead cleanup DNA extraction protocol (see Commit b4384aa: https://phyletica.org/lab-protocols/extraction-spri.html). DNA integrity was manually inspected on agarose gels, and concentrations were determined on a NanoDrop spectrophotometer and a Qubit fluorometer. Whole-genome DNA library preparation was performed using the Illumina DNA Prep library kits aiming at an insert size of ~350 bp. The resulting libraries were sequenced using 150-bp paired-end sequencing on Illumina NovaSeq S4 and SP instruments at the Harvard University Bauer Core and on a DNBSEQ-T7 instrument (Innomics Inc., Sunnyvale, CA; see table S1). To our new data, we added previous whole-genome resequence data of seven *H. charithonia* individuals and 36 genomes of 26 closely related species (table S1).

### Read mapping and genotype calling

Reads were filtered for adapters using Trimmomatic v0.39 (*94*) and mapped to the *H. erato demophoon* reference genome (*95*) using bwa-mem v0.7.15 (*96*) with default parameters. Median coverage across all *H. charithonia* samples was 13.0× (ranging from 4.6× to 126.0×; table S1). Genotyping was carried out with bcftools v1.17 mpileup and call modules, using the multiallelic-caller model (call -m), requiring minimum base and mapping qualities of 20. Genotypes were filtered using the bcftools filter module. Both invariant and variant sites were required to have a minimum quality score (QUAL) of 20. Furthermore, individual genotypes were filtered to have a depth of coverage (DP) ≥ 4 (except for the Z chromosome of females for which the minimum required DP ≥ 2) and QUAL ≥20. Genotypes not fulfilling these requirements or within 5 bp of an indel (--SnpGap) were recoded as missing.

### Population structure and phylogenetic analysis

Population structure was investigated using a PCA as implemented in PLINK v1.9 (*97*). We considered only *H. charithonia* individuals and only autosomal biallelic sites (excluding singletons) with no missing genotypes and each at least 25 kb from the next SNP.

We estimated phylogenies using all *H. charithonia* genome sequences and one of the sister species, *H. peruvianus*. Autosomal and Z-chromosome phylogenies were generated separately, using both variable and invariable sites. In PLINK, positions were selected to be at least 1 kb apart (--bp-space 1000) with no missing genotypes (--geno 0) across all 76 *H. charithonia* individuals and the *H. peruvianus* outgroup. For the Z chromosome, we select sites every 100 bp to retain more sites. The resulting variant call format (VCF) files were converted to FASTA format using a custom script (https://github.com/FernandoSeixas/Hcharithonia-inversions). We inferred ML trees using IQ-TREE v2.1.0 (*98*). Model selection was performed using ModelFinder (*99*) (-m MFP), and branch support was estimated using ultrafast bootstrap implemented in IQ-TREE (*100*), with 5000 ultrafast bootstrap replicates (-B 5000). To retain information at heterozygous sites, we assigned International Union of Pure and Applied Chemistry (IUPAC) ambiguity codes to which IQ-TREE assigned equal likelihood for each underlying base identity. To infer population relationships while accounting for admixture events, we estimated admixture graphs, using the “qpgraph” function of the ADMIXTOOLS 2.0.0 R package (*101*). Only autosomal SNPs with minimum allele frequency (MAF) ≥5%, with no missing genotypes (--geno 0) and at least 1 kb apart (--bp-space 1000) were considered, resulting in 247,944 SNPs. We ran the analysis grouping individuals by their assigned population (see table S1). Given the lack of population structure within *H. c. vazquezae* and to simplify this analysis, we included only a subset of *H. c. vazquaze* populations—Panama, Honduras, Mexico (two populations), and Texas. We considered admixture graphs with up to four admixture events, each estimated using five independent replicate runs. In each run, the best-fit graph was estimated using the command “find_graphs” with default parameters and specifying *H. peruvianus* as outgroup. For each number of admixture events, only the best run (i.e., the run with the lowest score) was considered. We then determined which model was best supported, by running 100 block-bootstrap replicates of the best graph under each model and comparing the likelihood score distributions using the “qpgraph_resample_multi” and “compare_fits” functions. Confidence intervals for strength of drift and admixture proportion were estimated using the “qpgraph_resample_snps” with 100 bootstraps.

We estimated a dated phylogeny for the mitochondrial genome. Whole mitochondrial genome sequences of each individual were assembled from a subset of 5 million trimmed reads with MITObim v1.9.1 (*102*), using the --quick option and up to 40 iterations. The full mitochondrial genome of *Heliconius sara* was used as bait (Genbank accession NC_026564). Mitochondrial genome assemblies were aligned using MAFFT (*103*) and pruned manually in Geneious version 2023.2.1 (*104*). We selected only genic regions for phylogenetic analysis, on the basis of annotations of the *H. sara* reference. Models of DNA evolution for each gene alignment were fit using ModelTest-NG (*105*, *106*). Bayesian phylogenetic inference in BEAST v2.6.3 (*107*) was used to date divergence times. Three independent runs of 10 million generations were performed using the best-fit nucleotide substitution model (or the next-most simple model implemented in the software), a BSP tree prior, and a strict molecular clock. Runs were examined in Tracer v1.7.1 for convergence and consistency across runs. Replicate runs were concatenated using LogCombiner, and postburn-in trees were summarized using TreeAnnotator, both part of the BEAST package. Node ages were calibrated assuming a substitution rate of 1.15 × 10^−8^ substitutions/site per year for the cytochrome oxidase subunit 1 (*COI*) region (*25*). We also constructed a median-joining network using PopART 1.7 (*108*, *109*).

### Summary statistics and genomic differentiation

Within-population (π) and between-population (*d_XY_*, Hudson’s *F_ST_*) summary statistics were estimated in 50-kb nonoverlapping sliding windows along the genome using the python script popgenWindows.py (available from github.com/simonhmartin/genomics_general). Sites with less than 80% individuals genotyped were discarded; only windows with at least 20% sites passing filters were considered. We found that the Jamaican population retains variation from an ancestral colonization wave of the Caribbean. To explore heterogeneity in ancestry across the genome, stemming from multiple colonizations of the Caribbean, we used Twisst (*110*) (https://github.com/simonhmartin/Twisst). Phylogenetic relationships were estimated among three focal subspecies [*H. c. simulator* (Jamaica), *H. c. churchii* (Dominican Republic), and *H. c. bassleri* (Colombia and Peru)], using *H. peruvianus* as the outgroup. Only SNPs variable in the focal species and without missing data were considered. Statistical phasing and imputation were performed using Beagle v5.1 (*111*), with default settings. ML trees were inferred from the phased filtered dataset, in 50-kb nonoverlapping windows, assuming a general time reversible (GTR) substitution model, in PhyML (*112*). Exact weightings were computed for all phylogenies.

### Genetic crosses

Captive-bred populations of *H. charithonia* from Florida and Texas were reared in the OEB Harvard greenhouses. Adult butterflies were fed with a solution of water with sugar and pollen and provided additional pollen sources—*Lantana* spp. (Verbenaceae). We performed crosses between captive bred *H. c. vazquezae* (Texas) with *H. c. tuckeri* (Florida). F_1_ individuals were obtained by crossing 1 pure Texas virgin-female with 1 pure Florida male, producing 32 viable adult offspring (16 females and 16 males). Three F_1_ female-male pairs were mated to generate F_2_ progeny (67 females, 64 males). Females were kept isolated from males before the crosses to ensure all were unmated. Individuals were monitored daily and stored once adult butterflies emerged.

### Historical demography and range expansion

Past demographic dynamics of *H. charithonia* were estimated using the PSMC model (*113*). Diploid consensus sequences were obtained using samtools v1.17 mpileup for all autosomal contiguous scaffolds longer than 1 Mb and requiring a minimum base and mapping quality of 20 and DP ≥8. Because of PSMC’s limitations inferring coalescent rates in the more recent past, we also used coalescent BSP (*114*) in BEAST for the mitochondrial genome, as described above. Since the *H. charithonia* mitochondrial lineage from Jamaica represents a basal and divergent lineage, the Jamaican mitogenomes were excluded from this analysis. The Bayesian Skyline demographic profile was generated in Tracer v1.7.1 and plotted with R.

We tested for range expansion of *H. charithonia* versus equilibrium isolation-by-distance using the method outlined in (*26*). This method relies on allele frequency clines created by successive founder events during a range expansion and can infer the strength of the founder effects associated with spatial expansion and the most likely expansion origin (*26*, *115*). The data were prepared using PLINK, considering only SNPs with no missing data and a MAF of 0.05 and at least 10 kb apart. The *H. peruvianus* individual was included to determine the derived state of each allele. The filtered dataset was then analyzed in the R rangeexpansion package (*26*). This analysis was performed both including all individuals and excluding individuals from Jamaica.

### Detection of divergent haplotypes

To identify genomic regions with outlier population structure, we performed local PCA with the lostruct R package (*44*). We used the dataset including only *H. charithonia* with biallelic sites (excluding singletons) with a maximum of 5% missing genotypes. Local PCA in lostruct was performed for nonoverlapping windows of 500 SNPs and independently for each chromosome using the eigen_windows function. The distance matrix between windows from local PCs was then computed using the pc_dist function (with the two top PCs) with default parameters and distances were visualized using multidimensional scaling (MDS) with the cmdscale function with two MDS axes. The two MDS axis were then visualized by plotting the MDS score against the genomic position of each window. The *z*-score of the MDS1 score for each window was calculated; potential haploblocks of interest corresponded to genomic regions with at least five consecutive windows with a *z*-score > 3.

All SNPs within these haploblocks were used to calculate PCAs using PLINK. After inspecting the PCAs, samples were manually assigned to groups, as in every haploblock the individuals segregated into well-defined clusters. For each region, we also measured heterozygosity for every sample (reported as the percentage of called genotypes within that sample that are heterozygous) using bcftools.

For each chromosome harboring a putative inversion, we estimated pairwise linkage disequilibrium (*r*^2^) considering either (i) all *H. charithonia* individuals or (ii) only individuals of the most represented cluster as defined by the PCA of the region. In this analysis, we excluded two individuals with more than 20% missing data. Only biallelic SNPs with MAF > 0.05 and at least 1000 bp apart were considered. Genotype *r*^2^ values were calculated with vcftools geno-r2 (*116*). Last, the mean *r*^2^ values were calculated between all 100-kb windows within a chromosome were calculated using the script emerald2windowldcounts.pl (from https://github.com/owensgl/reformat).

To measure genetic differentiation between standard and inversion haplotypes, we calculated absolute sequence divergence (*d_XY_*) using the python script popgenWindows.py (available from github.com/simonhmartin/genomics_general). This was calculated in 50-kb nonoverlapping sliding windows along chromosomes harboring the inversions, between the predicted homozygote genotypes.

To determine whether divergent haplotypes correspond to inversions, we compared the chromosome level genome assemblies of one *H. c. vazquezae* individual from Texas (*117*) and one *H. c. charithonia* from Puerto Rico (*118*) to the two *H. erato* (*95*, *119*), the *H. sara* (*120*), and the *H. melpomene* (*121*) reference genomes. Identification of SVs was performed using SyRI 1.6.3 (*122*). SyRI expects chromosome-level assemblies with the same number of chromosomes. Hence, for the genome assemblies of *H. melpomene*, *H. erato demophoon*, *H. e. lativitta*, and *H. charithonia* from Puerto Rico, we concatenated scaffolds in the same chromosome. Pairwise genome-to-genome alignments were then performed using minimap2 (*123*), with parameters “-ax asm20 –eqx,” and SVs were identified using SyRI, with parameters “-f -k.” We also mapped the Hi-C data used to generate the Texas *H. charithonia* genome assembly to both the Texas and the Puerto Rico *H. charithonia* genomes. Note that the Hi-C data come from an individual (not included in our population genomics dataset) descendant from that used to produce the PacBio data, but both are homozygotes for the chromosome 19 inversion. Mapping of Hi-C data to the two genomes was carried using HiC-Pro v3.1.0 (*124*) with default parameters, and the contact maps were visualized using pyGenomeTracks (*125*, *126*).

### Timing the origins of inversions

To reconstruct the evolutionary history of the inversions, we analyzed an extended dataset including 18 closely related species (26 subspecies; table S1). Read mapping and genotype calling were performed as described above. Absolute genetic distance (*d_XY_*) between both the standard and inversion haplotypes and the outgroups was calculated in sliding windows of 50 kb (50-kb step) along chromosomes using the python script popgenWindows.py (available from github.com/simonhmartin/genomics_general).

Phylogenetic relationships within the inversion regions were estimated on the basis of ML concatenated gene trees using IQ-TREE (*98*). *H. charithonia* individuals, homozygous for each inversion haplotype, and individuals representative of the different *Heliconius* clades were included. Sites without missing information in all individuals were considered. Model selection was performed using ModelFinder (*99*) (-m MFP), and branch support was estimated using ultrafast bootstrap implemented in IQ-TREE (*100*), with 5000 ultrafast bootstrap replicates (-B 5000). To retain information at heterozygous sites, we assigned IUPAC ambiguity codes to which IQ-TREE assigned equal likelihood for each underlying base identity. Phylogenetic relationships across the genome were also estimated for *H. charithonia* harboring different inversion genotypes, as well as outgroup species, using the multispecies coalescent approach implemented in BPP v.4.6.2 (*127*). Only a subset of species was considered, and *H. melpomene* was used as an outgroup. Loci were selected to be 300 bp long, at least 2 kb apart from the nearest loci and at least 2 kb apart from exons as annotated in the reference genome. Repetitive elements as annotated in the reference genome were masked before producing sequence alignments. For each locus, individuals with more than 50% missing genotype calls were excluded from the alignment, and only loci with at least two individuals per population were considered. Furthermore, sites with more than 20% of individuals with missing genotype calls were removed, and loci with less than 50 bp passing filters were excluded. Loci were grouped into blocks of 100 loci, and those overlapping the inversions on chromosomes 2, 6, and 19 were grouped in separate blocks. Species-tree estimation was then performed in BPP using the A01 analysis (species-tree inference assuming no gene flow). Inverse gamma priors (invGs) were applied both to the root age (τ0) and to effective population sizes (θ)—invG (3, 0.3) and invG (3, 0.04), respectively. Parameters were scaled assuming a mutation rate of 2.9 × 10^−9^ substitutions per site per generation and a generation time of 0.25 years (*24*). The Markov chain Monte Carlo was run for 1,000,000 iterations after 32,000 iterations of burn-in, sampling every 2 iterations.

To estimate divergence times between inversion haplotypes, we again used BPP (*127*), but assuming a fixed species tree (A00 analysis). For each inversion, the inversion topologies inferred from the BPP A01 analyses were used. We also estimated divergence times between species, using the collinear parts of the genome. For this, we assumed the majority tree across all noninversion blocks to be the true species tree. To reduce the amount of data, we subsampled 10 loci from each noninverted blocks in chromosome 2, 6, and 19. The analysis were run using the same priors, burn-in, and number of iterations as in the BPP A01 analyses.

### Functional impact of inversions

We used the *H. erato demophoon* gene annotation (*95*) to explore whether inversion breakpoints disrupted annotated genes. The coordinates of inversion breakpoints on chromosome 2 were determined on the basis of the local PCA results and genome alignments, while for the chromosome 6 and 19, we relied on the local PCA results. For each inversion, we recorded genes spanning the breakpoints or the closest annotated gene to the left and right of the inversion breakpoint.

To test whether inversions are enriched for deleterious mutations, we calculated the ratio of nonsynonymous to synonymous polymorphisms (*pN*/*pS*) within *H. charithonia*, the ratio of synonymous to nonsynonymous substitutions (*dN*/*dS*) compared with *H. e. demophoon*, and the DoS (*128*). SNPs in *H. charithonia* were annotated using SNPEff v5.1 (*129*), with default parameters, and the *H. e. demophoon* reference genome annotation. To ensure each gene comprises several SNPs, only genes larger than 5 kb were considered. These metrics were calculated for each inversion region using only individuals homozygous for each of the inversion haplotypes, while whole-genome distributions were obtained using all individuals and calculated on 500-kb nonoverlapping windows.

To assess whether heterokaryotypes are maintained by selection—potentially as a consequence of masking mutational load—we tested HWE for inversion genotype frequencies within *H. c. vazquezae* (the only subspecies in which inversions are polymorphic), using HWE.chisq in R from the “genetics” package.

To identify SNPs associated with environmental variables, we used latent factor mixed models, as implemented in the LEA R package (*130*–*132*). This analysis tests for significant associations between SNP allele frequencies and the selected environmental variables after correcting for genetic structure. SNPs were filtered to include only those with no missing data, a MAF of 0.05, and at least 100 bp apart from each other, resulting in 1,088,130 SNPs. The climatic variables were obtained from the WordClim database, with 2.5 arc min (~4.6 km^2^) resolution (*133*), and extracted for each specimens’ location using QGIS 3.36.2. Genotype-environment (GE) associations were inspected using the *lfmm2* function, with the lambda default parameter of 1 × 10^−5^, and number of factors K = 3 to account for population structure. The resulting *P* values were adjusted to account for multiple testing using the Benjamini-Hochberg method. The same GE tests were performed but coding the inversion as a single locus and selecting SNPs at least 10 kb apart from collinear parts of the genome.

### Gene flux within inversions

To detect and quantify possible gene flow between inversion haplotypes, we fitted and compared strict divergence (DIV) and Isolation-with-Migration (IM) models using gIMble v1.0.3 (*134*). Two individuals per inversion haplotype were included in the analysis. A VCF containing genotype calls only for the individuals included in this analysis was first produced using freebayes v1.3.10 (*135*), considering only reads with a minimum mapping quality of 20 and bases with a minimum quality of 20. Only genotype calls with a minimum depth of 8 and SNPs with a quality of ≥20 were considered. The VCF was next preprocessed using gimbleprep. Since, demographic models fit by gIMble assume a neutral model of evolution, we focused only on intergenic regions. We then ran the gIMble parse module for all possible haplotype pairwise comparisons within each inversion region, to quantify genetic diversity and divergence for the filtered subset of the data. We summarized variation in pair blocks of 64 bases and tallied all blockwise site frequency configurations of mutations across pairs, using gIMble blocks and gIMble tally modules. Last, we fitted “strict divergence” (DIV) and “isolation with migration” (IM) demographic models to using the gIMble optimize module. gIMble query was run to extract model summary information, including composite likelihood, for each model allowing us to determine the best fitting of the three models for each of inversion regions and inversion genotype pairwise combinations.

To explore possible gene flux between nonrecombining haplotypes at a finer scale along inversion regions, we also estimated phylogenetic relationships in sliding windows among sets of individuals homozygous for each inversion haplotype using Twisst. For the chromosome 2 inversions, we considered four groups: three Continental populations—homozygous for the standard haplotype (g00_cont), homozygous for the larger inversion haplotype (g11_cont), and homozygous for the nested inversion haplotype (g22_cont)—and the Jamaican population in which all individuals are homozygous for the larger inversion haplotype (g11_isld). For inversions on chromosome 6 and 19, we considered three populations: two Continental—homozygous for the standard haplotype (g00_cont) and homozygous for the inversion haplotype (g11_cont)—and the Jamaican population in which all individuals are homozygous for standard haplotype (g00_isld). One *H. erato* individual was used as the outgroup. Gene flux between inversion haplotypes is expected to result in phylogenies grouping Continental populations carrying different haplotypes, while in the absence of gene flux populations in the Continent and in the Caribbean carrying the same haplotype should be sister. Only SNPs variable in the focal species and without missing data were considered. Statistical phasing and imputation were performed using Beagle, with default settings. ML trees were inferred from the phased filtered dataset, in 25-kb nonoverlapping windows, assuming a GTR substitution model, in PhyML. Exact weightings were computed for all phylogenies.
