# Supplementary material for: Ancient inversion polymorphisms are locally adaptive in a widespread butterfly species
Source: Sci Adv. 2026 Jul 1;12(27):eadv1982. doi: 10.1126/sciadv.adv1982 (PMC13322245; doi:10.1126/sciadv.adv1982)
Supplement: Supplementary file 1 — Figs. S1 to S25 Legends for tables S1 to S5 [file sciadv.adv1982_sm.pdf]

Science Advances
AAAS

Supplementary Materials for

Ancient inversion polymorphisms are locally adaptive in a widespread butterfly species

Fernando A. Seixas *et al.*

Corresponding author: Fernando A. Seixas, fernandoferreiraseixas@gmail.com

Sci. Adv. **12**, eadv1982 (2026)
DOI: 10.1126/sciadv.adv1982

The PDF file includes:

Figs. S1 to S25
Legends for tables S1 to S5

Other Supplementary Material for this manuscript includes the following:

Tables S1 to S5

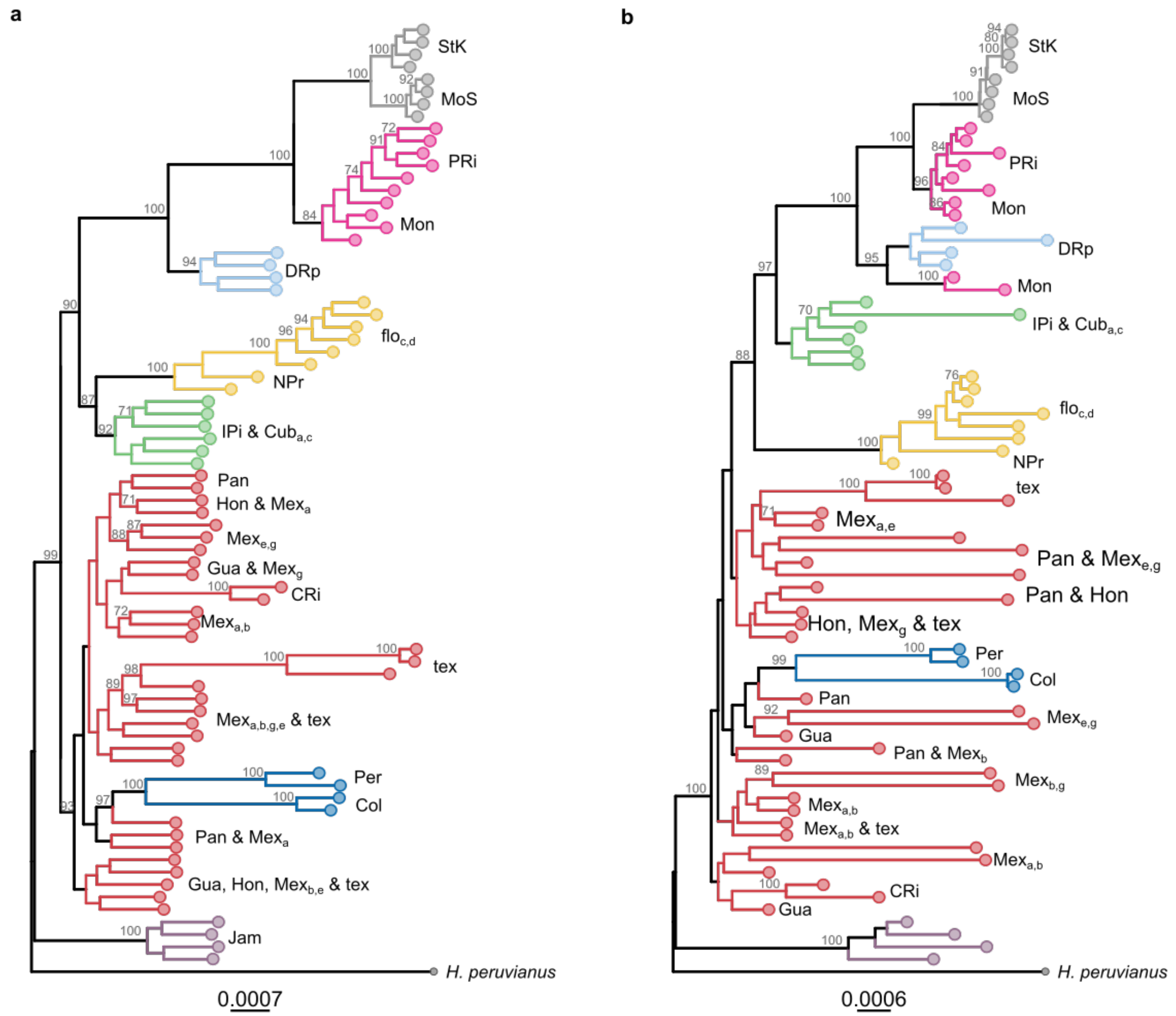

Fig. S1. **Autosomal and Z-chromosome phylogenetic trees.** Maximum-Likelihood (ML) tree based on **(a)** 267,524 autosomal sites sampled every 1-kb and **(b)** 73,399 Z-chromosome sites sampled every 1-kb and 100 bp, respectively.

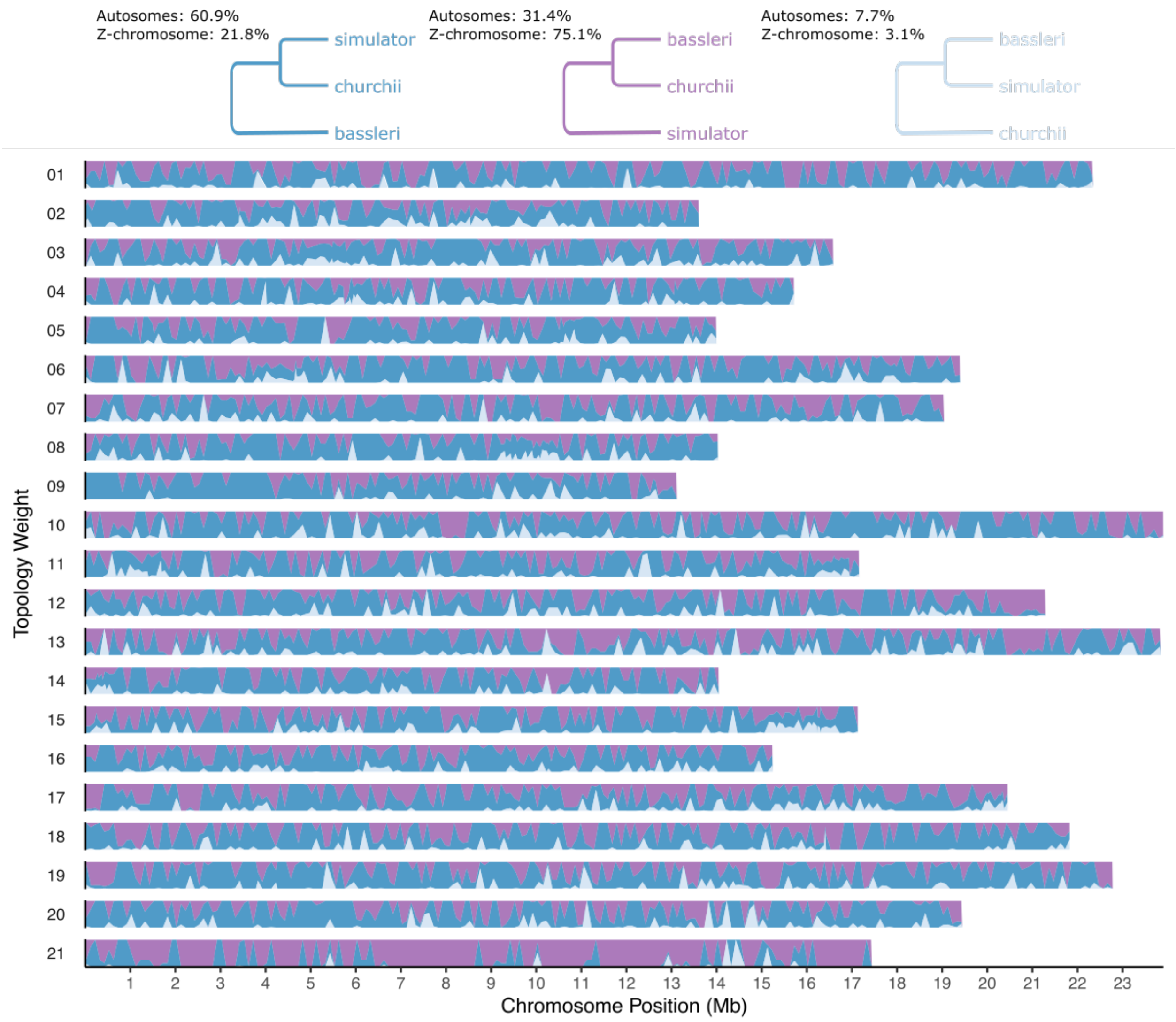

Fig. S2. Subspecies relationships along chromosomes. Topology weightings were estimated using Twisst, in non-overlapping 50 kb window and smoothed as a locally weighted average. The three possible topologies and their respective average weights across autosomal and Z-chromosome windows, are indicated on top.

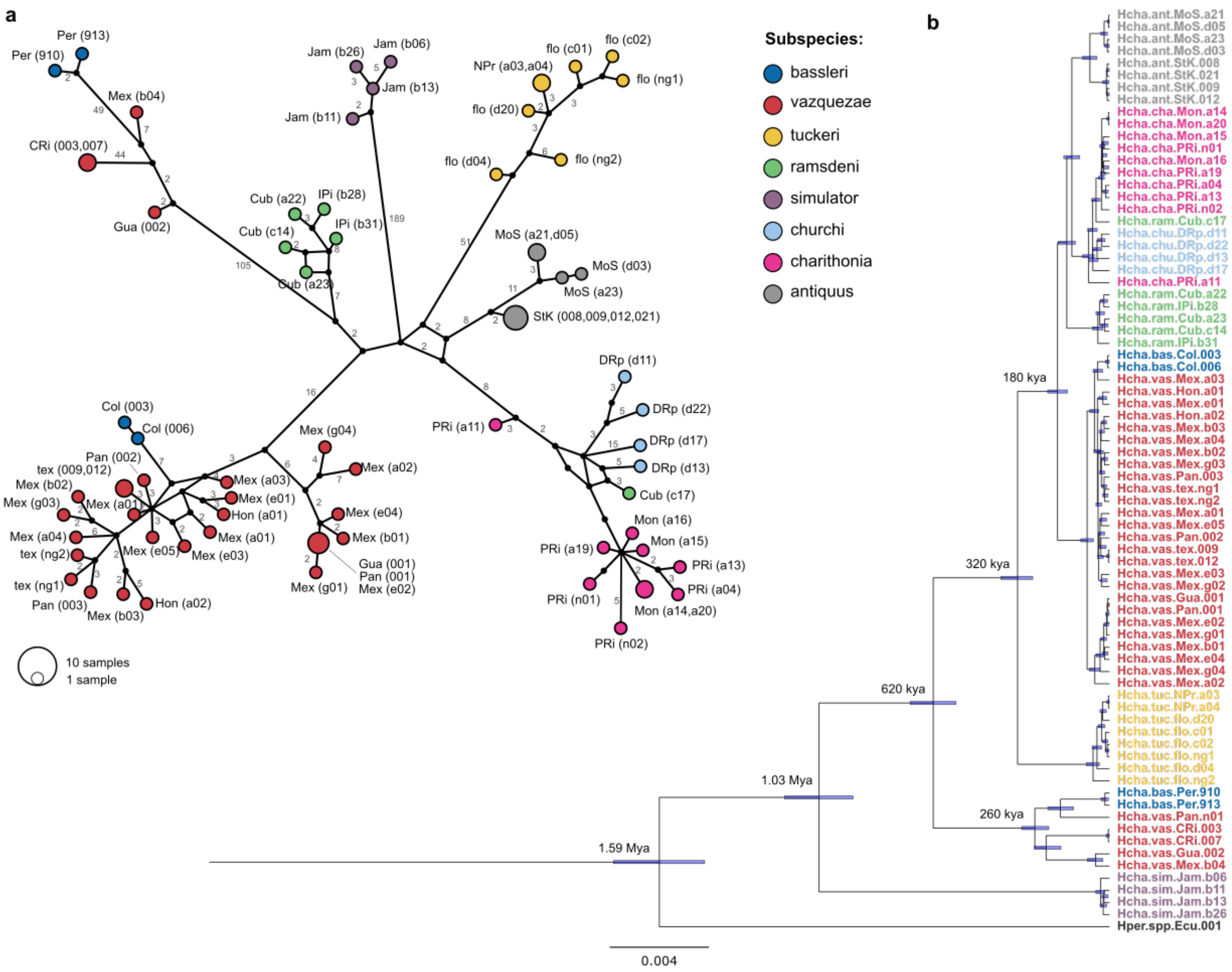

Fig. S3. **Mitochondrial haplotype network and Bayesian phylogenetic tree suggest at least two colonization waves of the Caribbean islands. (a)** Median-joining network of concatenated mitochondrial gene alignments. Circle sizes are proportional to the number of individuals with that same haplotype and are colored according to subspecies. Location codes and individuals' codes within these locations are provided next to haplotypes. Mutational steps along edges are shown except for edges with only a single mutation. **(b)** Mitochondrial Bayesian tree estimated in BEAST. Node ages are depicted in million years (Mya) and were calibrated assuming a substitution rate of 1.15×10^{-8} substitutions/site/year of the Cytochrome c oxidase subunit 1 (COI) region. Node bars indicate the 95% HPD intervals. Individuals are colored according to subspecies and their respective codes are provided in Supplementary Table 1.

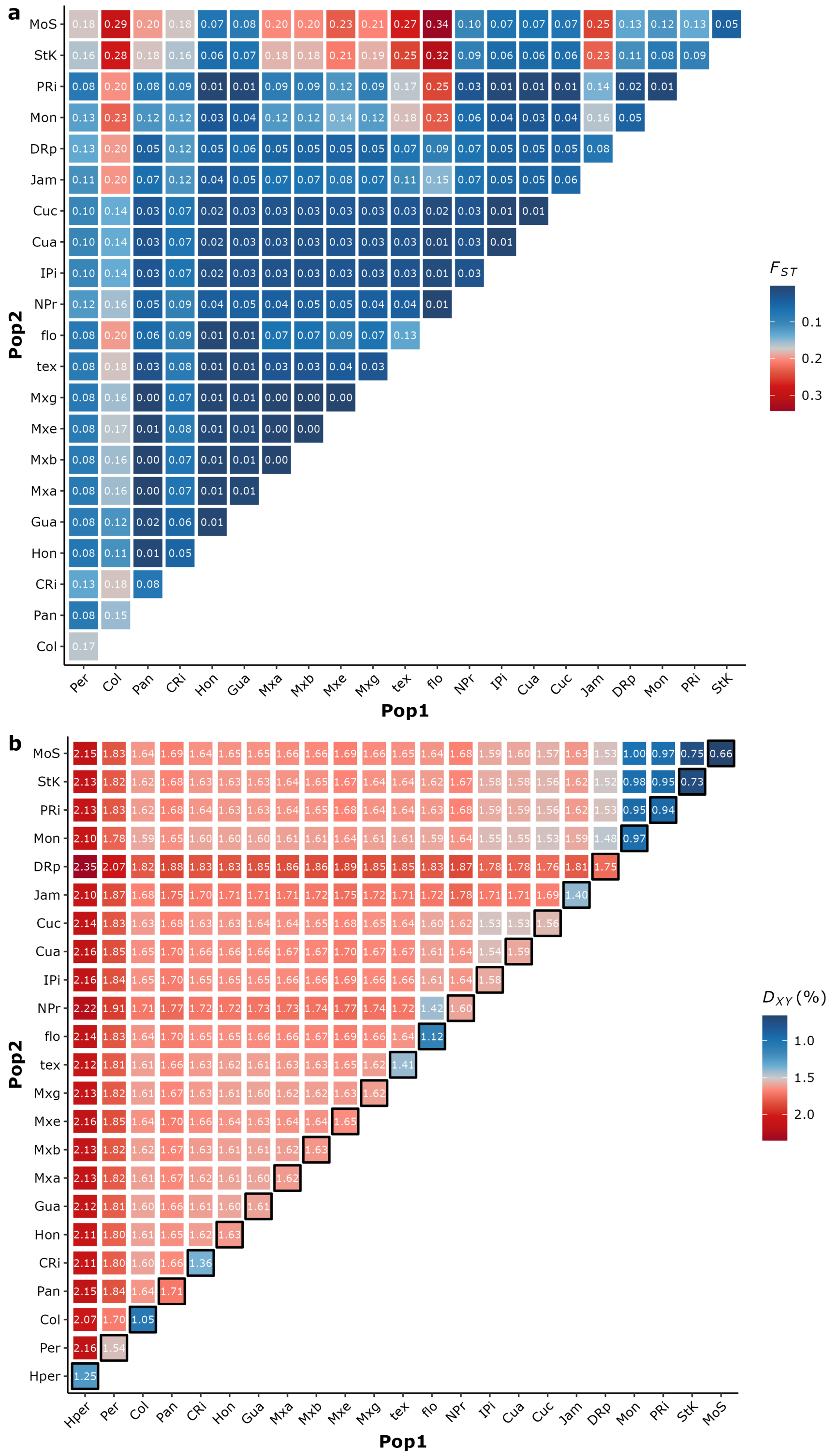

Fig. S4. Genome-wide levels of population divergence and diversity. (a) Mean genome-wide F_{ST}, calculated in 50 kb non-overlapping windows. **(b)** Mean genome-wide nucleotide diversity (π; diagonal) and pairwise population absolute divergence (d_{xy}; off-diagonal), calculated in 50 kb non-overlapping windows. Population codes are provided in Supplementary Table 1.

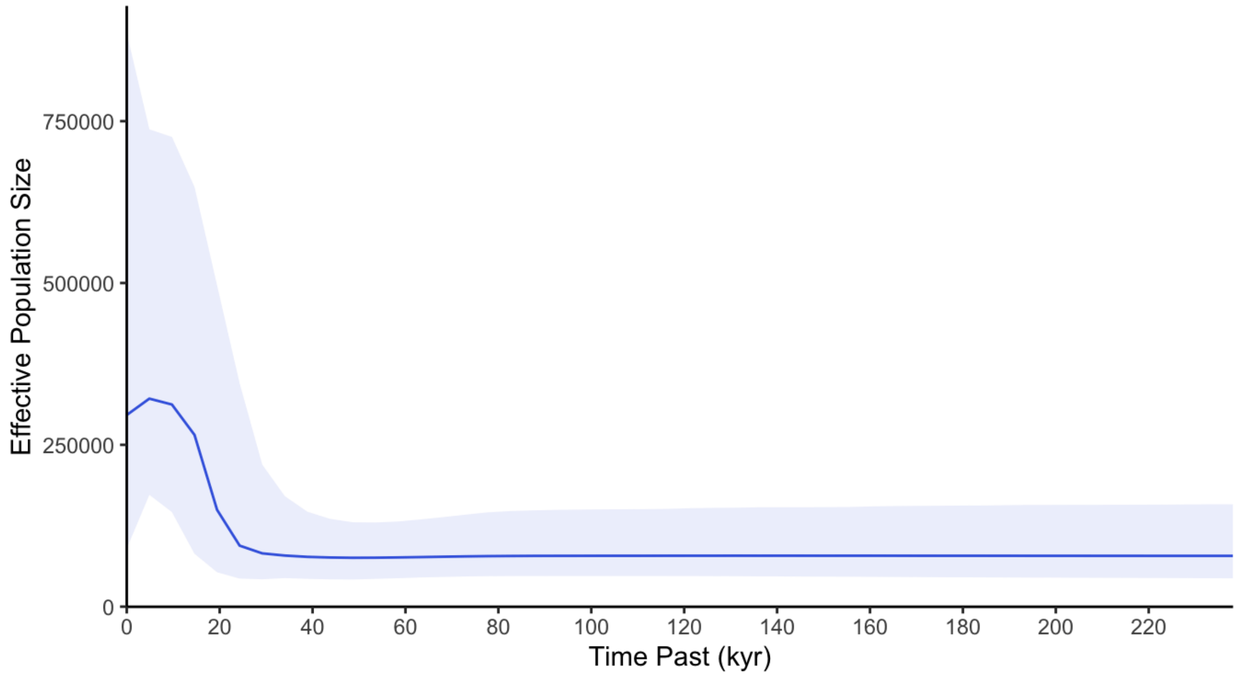

Fig. S5. **Bayesian skyline plot (BSP) of *H. charithonia* mitochondrial haplotypes.** Individuals from Jamaica were excluded. The solid blue line represents the median effective population size and the blue band the 95% high posterior density (HPD) interval. On the y-axis, the effective population sizes (N_e) are scaled to the mutation rate, whereas the x-axis represents coalescent time in thousands of years (kyr).

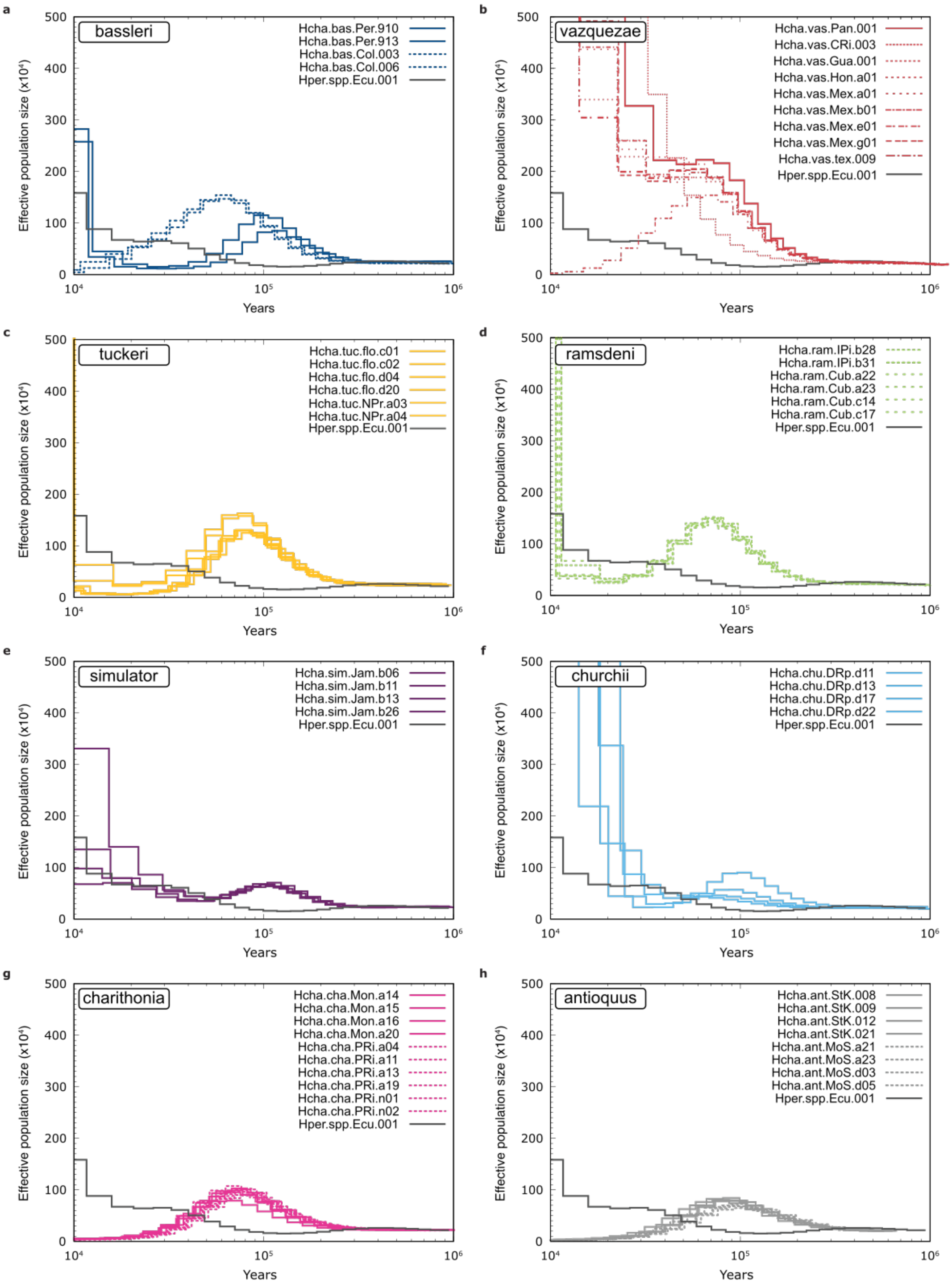

Fig. S6. Reconstruction of the past demographic history of *H. charithonia* using the Pairwise Sequentially Markovian Coalescent (PSMC) model from autosomal data. Times were

calibrated assuming a substitution rate of 2.9×10^{-9} substitutions/site/generation and a generation time of 0.25 years.

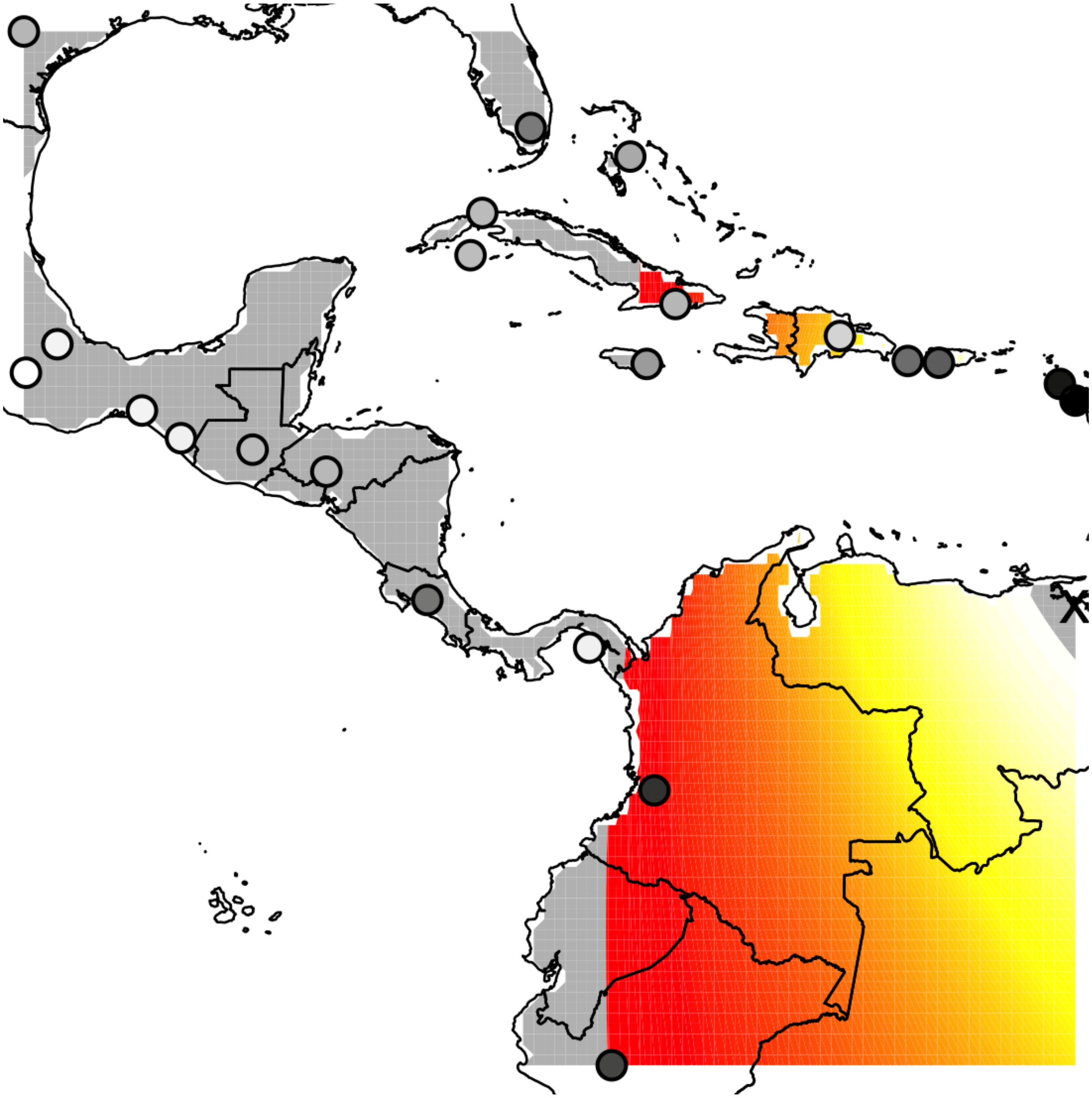

Fig. S7. Inference of a range expansion. The directionality index (ψ) detects a range expansion with origin in Venezuela (X mark). The likelihood of a region being the origin of the expansion is depicted by a gradient from yellow (less likely) to red (more likely), while gray regions indicate an unlikely origin. Each dot represents a sampled population and fill color indicates levels of diversity (darker grey indicates lower diversity).

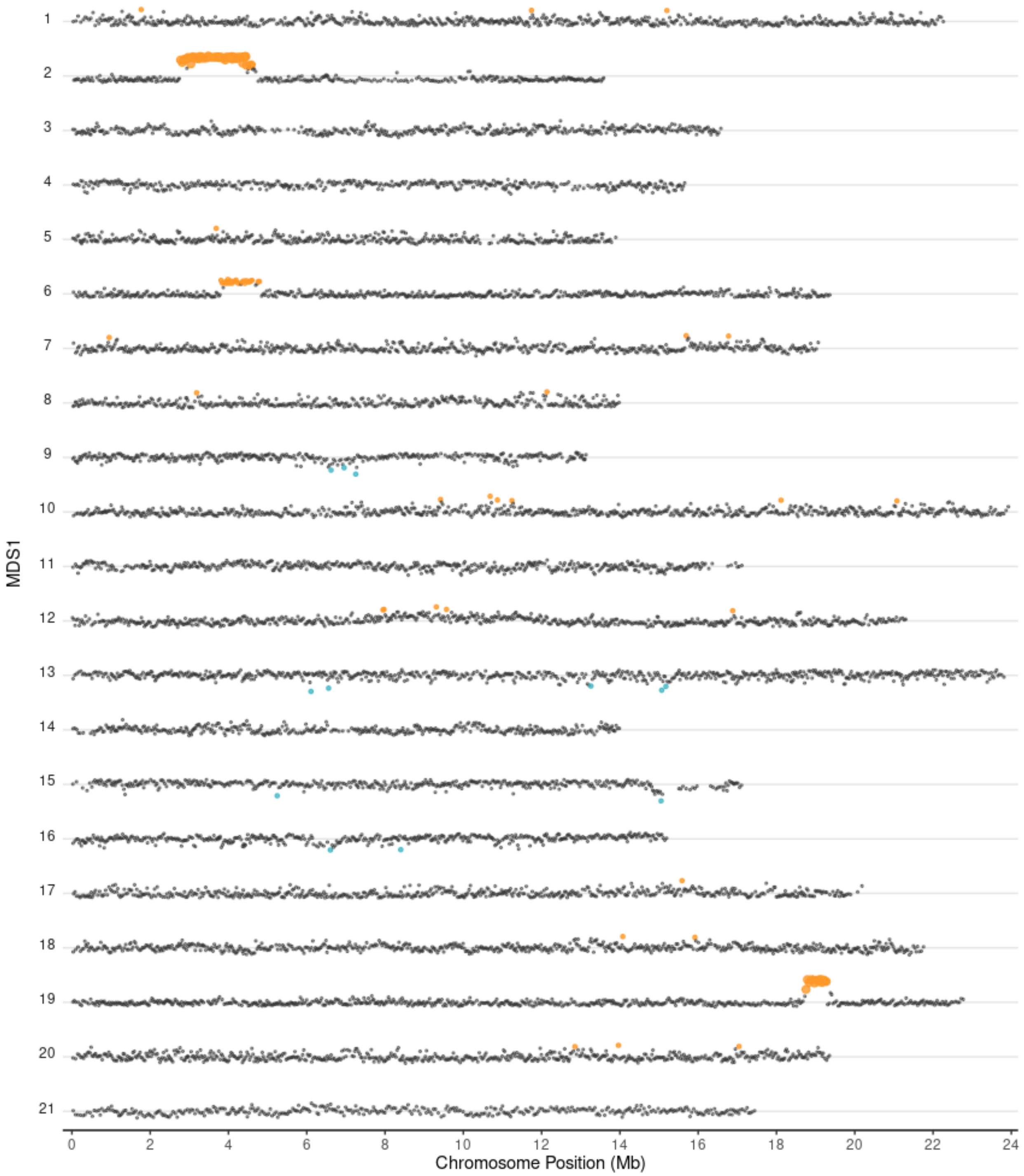

Fig. S8. Local PCA MDS plots along chromosomes. Each dot represents a 500 SNP window (median block size = 31,120 bp), and windows with outlier MDS scores as measured by z-score are highlighted in orange (z-score > 3) and blue (z-score < -3).

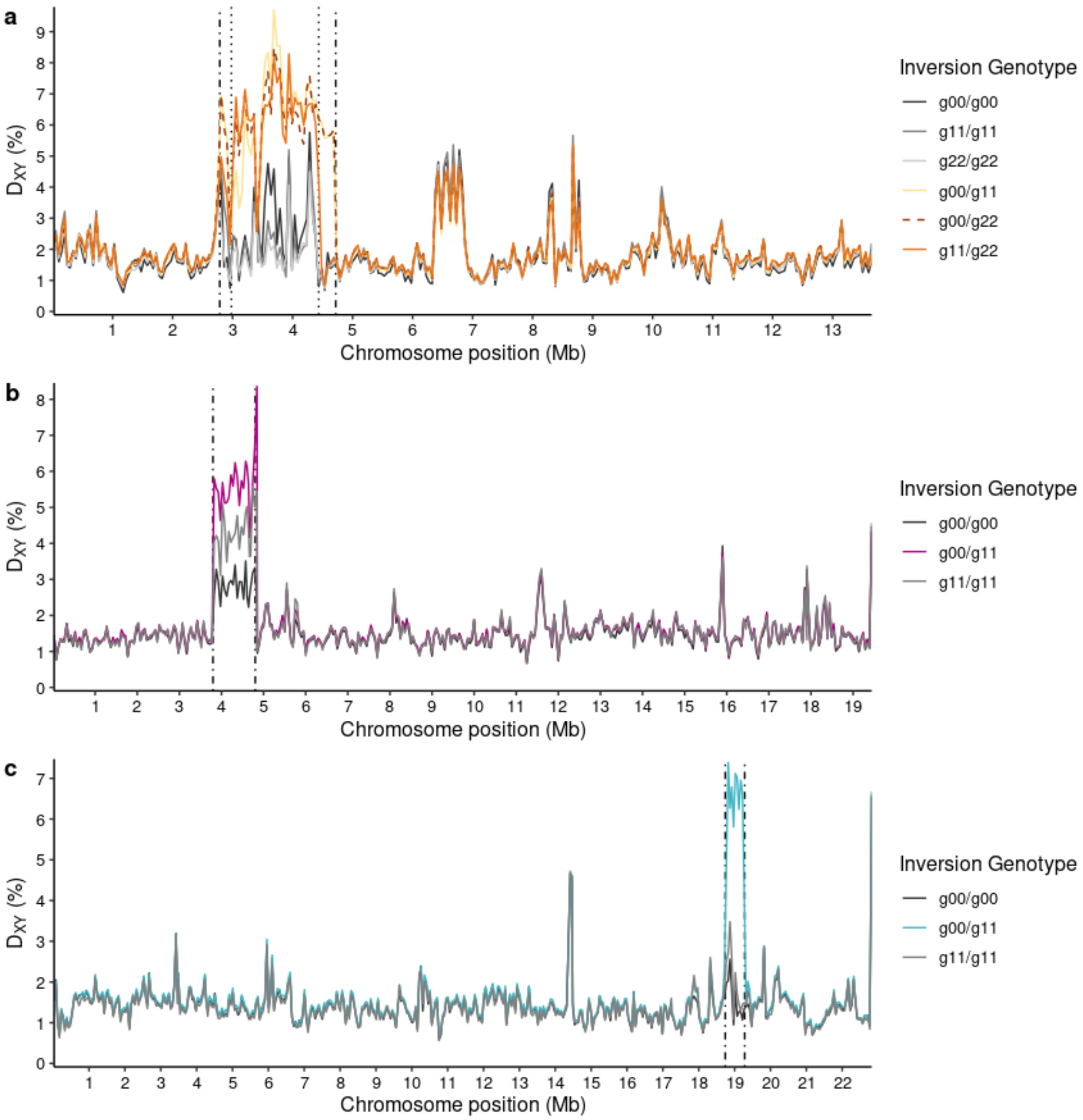

Fig. S9. Absolute divergence (d_{xy}) along chromosomes 2 (a), 6 (b) and 19 (c). d_{xy} was calculated in non-overlapping 50 kb windows, between individuals homozygous for different haplotypes at the putative inversions and within groups of individuals homozygous for the same haplotype (i.e., nucleotide diversity). Individuals' genotypes were defined following the PCA of the putative inversion regions (Figure 2D-F). The dashed-dotted lines indicate the putative inversion breakpoints according to the local PCA (Fig. S8; Figure 2A-C).

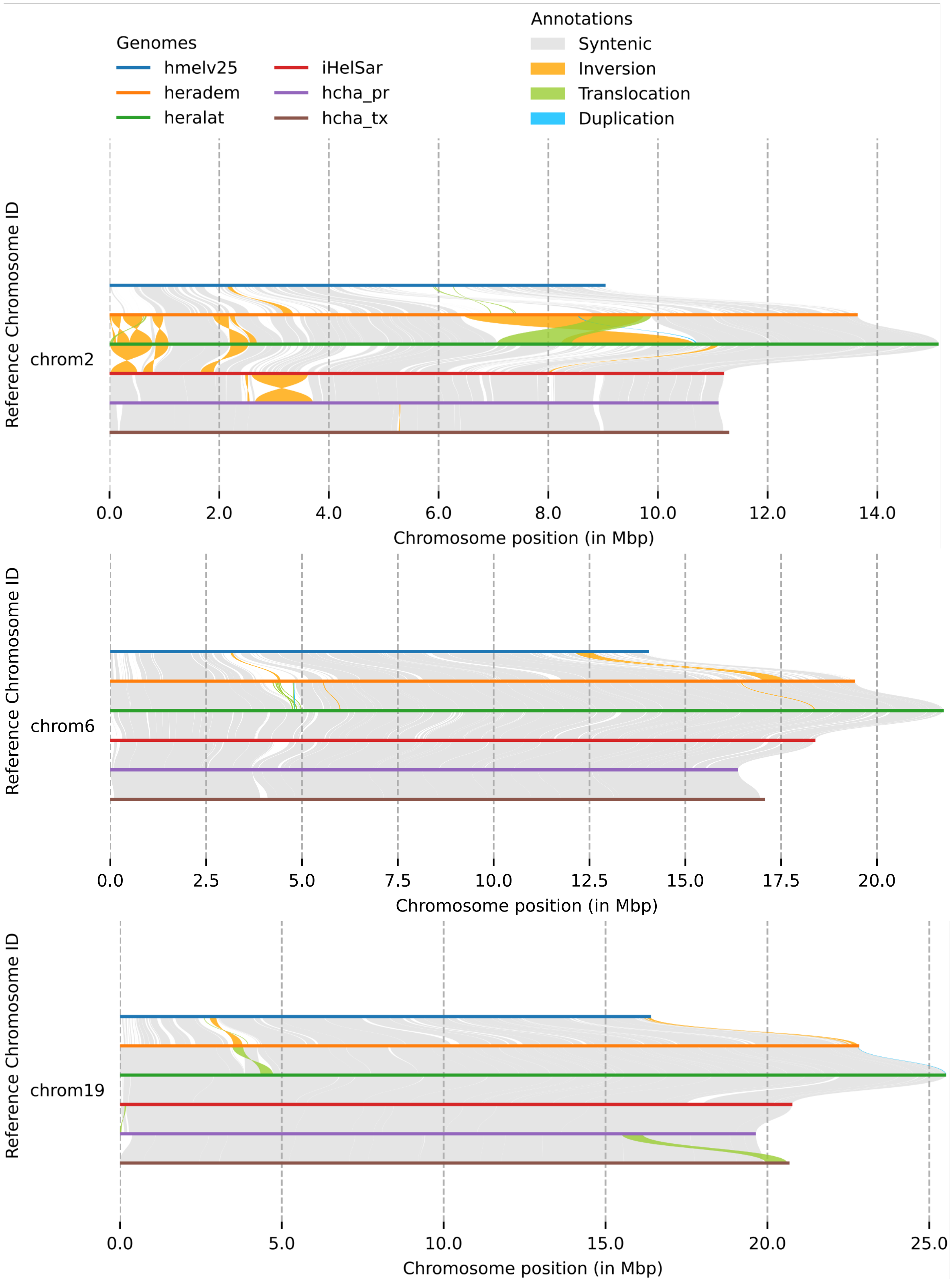

Fig. S10. Detection of structural rearrangements from chromosome alignments. Species codes: *H. melpomene* (hmelv25); *H. erato demophoon* (heradem); *H. erato lativitta* (heralat); *H. sara* (iHelSar); *H. charithonia* Puerto Rico (hcha_pr); *H. charithonia* Texas (hcha_tx)**.**

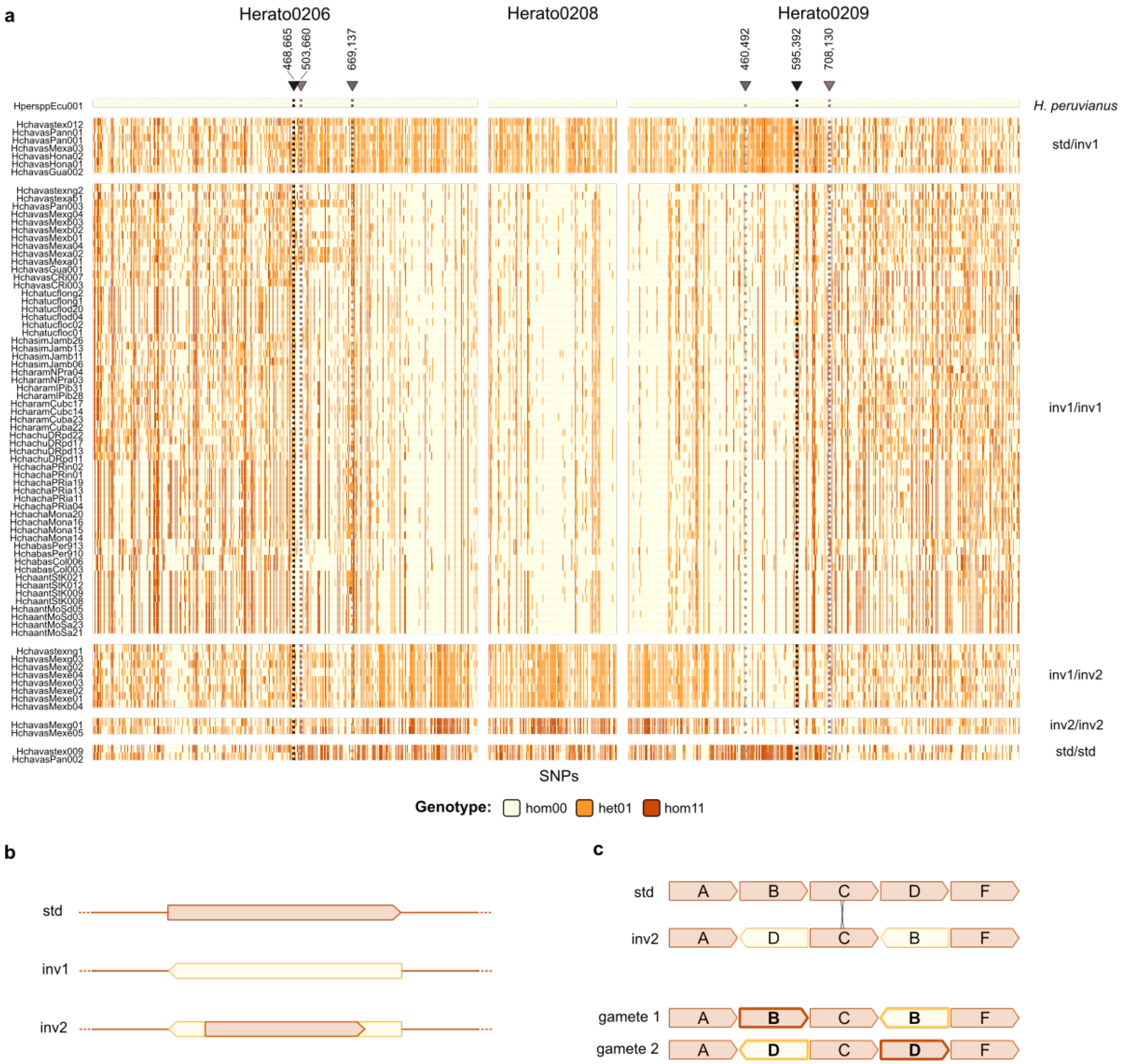

Fig. S11. In-depth analyses of the outlier region on chromosome 2. (a) Genotype plot for the region encompassing and surrounding the chromosome 2 outlier region. The inverted triangles and vertical lines indicate the coordinates of the putative inversion breakpoints according to the local PCA analysis (dark and light grey) and genome alignments (purple) – Fig. S8; Figure 2a. Note two pairs of possible breakpoint coordinates are given based on the local PCA, one more conservative (i.e. breaking the block if more that 2 consecutive windows are not outliers; light grey) and less conservative (i.e. allowing 2 consecutive non-outlier windows within the block; dark grey). On the y-axis, individuals are organized into their respective clusters as inferred by PCA of the chromosome 2 outlier region (Figure 2d). **(b)** Illustration of the hypothesized standard and inverted haplotypes. **(c)** Recombination between the standard and secondary inversion haplotypes, within their collinear region, can produce gametes carrying both deletions and duplications. The ancestral (std) and derived states (inv1, inv2) of the inversion region were determined based on genome alignment to other *Heliconius* (see Fig. 1M and Supp. Fig. 10).

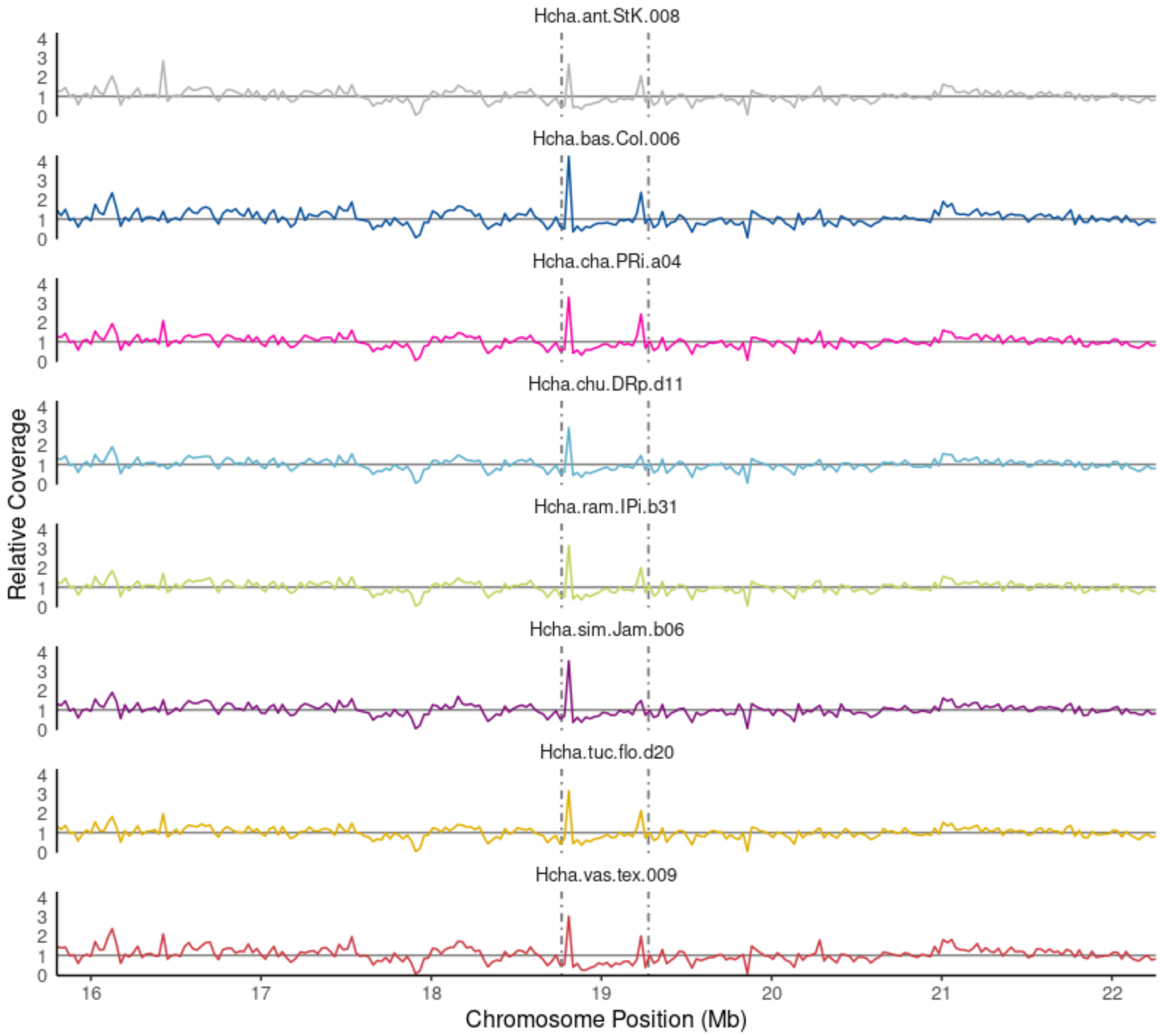

Fig. S12. Relative coverage along chromosome 19. Coverage along chromosome 19 was estimated based on reads mapped to the *H. erato demophoon* reference genome, in sliding windows of 50 kb. Relative coverage was obtained by dividing each window coverage by the median coverage across all autosomal windows. The dashed-dotted lines indicate the putative inversion breakpoints according to the local PCA (Fig. S8; Figure 2a,b). Individuals are colored according to their respective subspecies assignment (see Figure 1; Supplementary Table1).

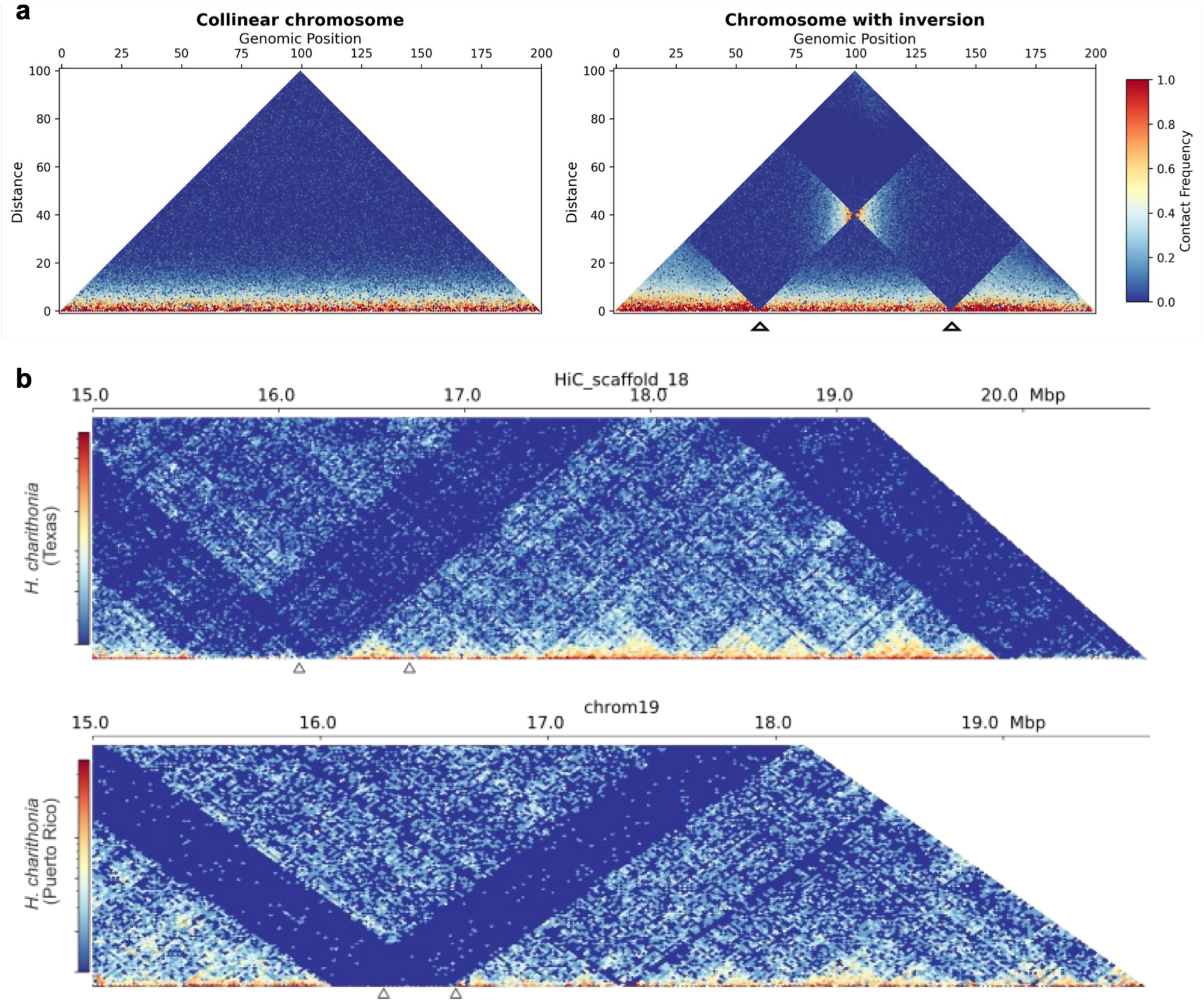

Fig. S13. Hi-C contact maps fail to directly confirm the putative inversion on chromosome 19. (a) Schematic of Hi-C contact maps for a chromosome without (left) and with an inversion (right), illustrating the typical "bowtie" pattern expected for inversions. **(b)** Hi-C data from a Texas individual (used to generate the Texas *H. charithonia* assembly) was mapped to both the Texas (top panel) and Puerto Rico (bottom) reference genome assemblies. The triangles indicate the approximate location of inversion breakpoints.

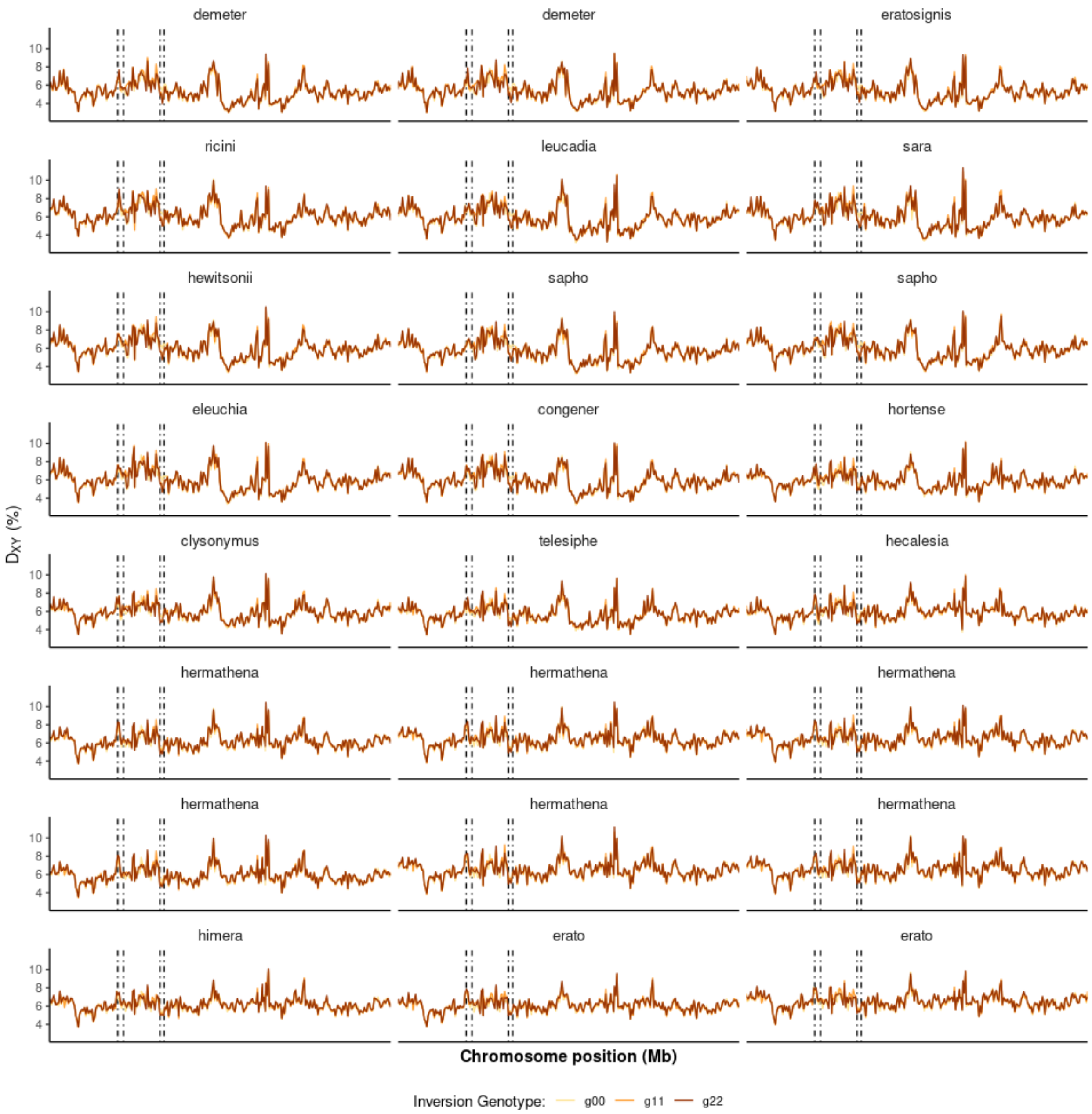

Fig. S14. Absolute genetic distance (*d_{xy}*) to outgroup species along chromosome 2 (50 kb non-overlapping windows). *d_{xy}* was calculated between outgroup species and either one *H. charithonia* individual homozygous for the inversion (dark brown) or one *H. charithonia* individual homozygous for the standard haplotype (orange). The dashed-dotted lines indicate the putative inversion breakpoints according to the local PCA and genome alignments (Fig. S8; Figure 2a).

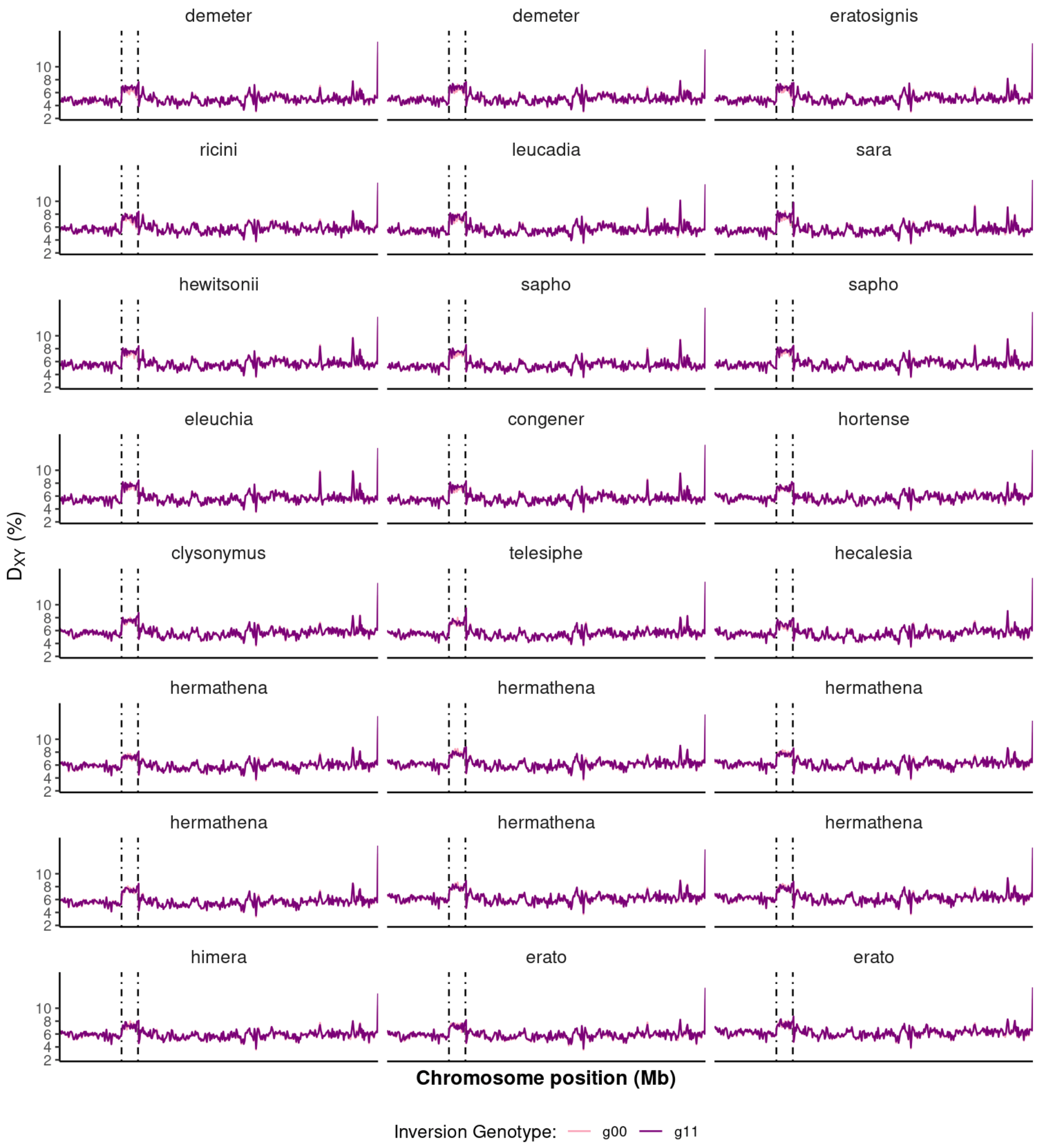

Fig. S15. Absolute genetic distance (*d_{xy}*) to outgroup species along chromosome 6 (50 kb non-overlapping windows). *d_{xy}* was calculated between outgroup species and either one *H. charithonia* individual homozygous for the inversion (dark purple) or one *H. charithonia* individual homozygous for the standard haplotype (pink). The dashed-dotted lines indicate the putative inversion breakpoints according to the local PCA (Fig. S8; Figure 2b).

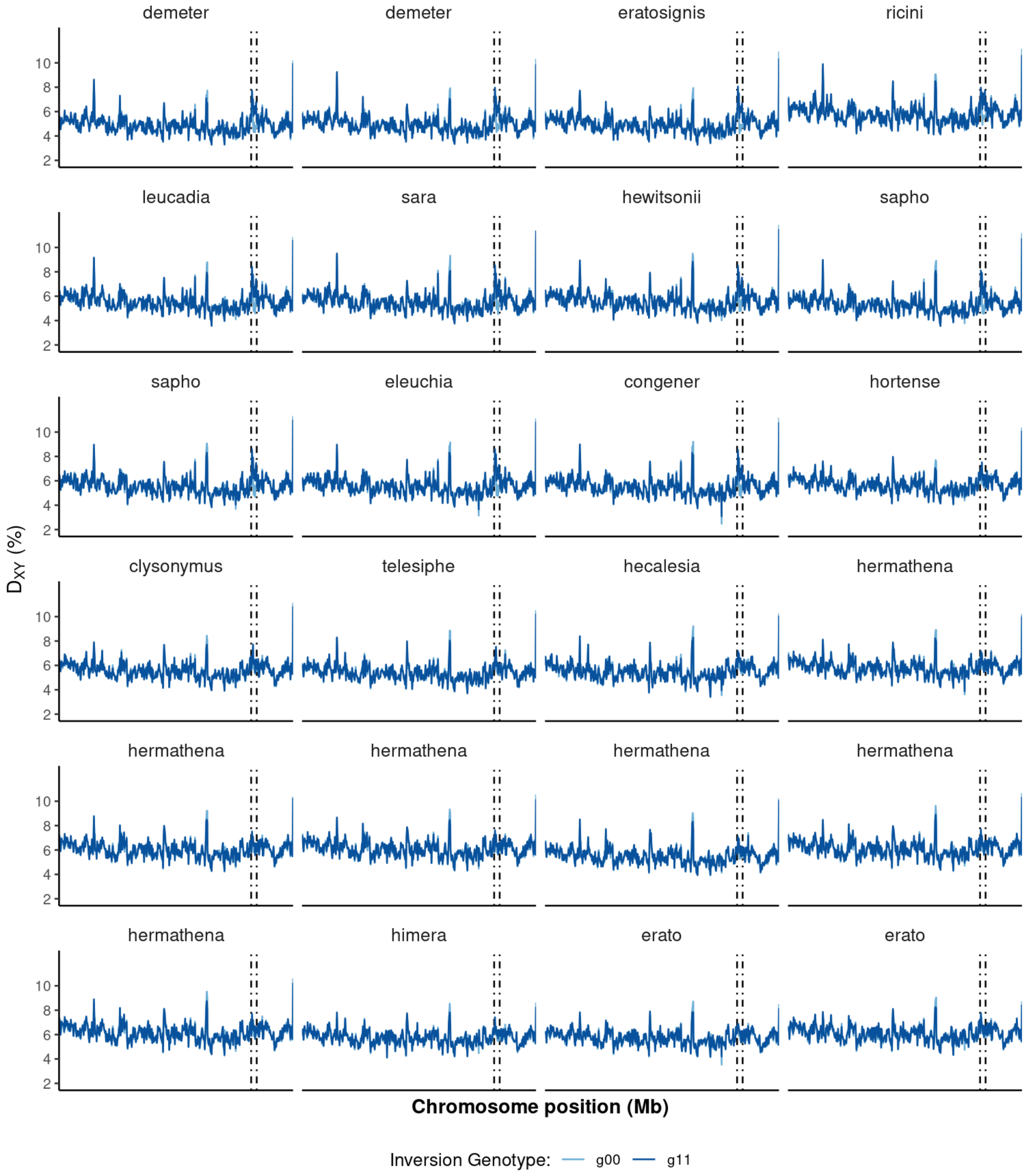

Fig. S16. Absolute genetic distance (d_{xy}) to outgroup species along chromosome 18 (50 kb non-overlapping windows). d_{xy} was calculated between outgroup species and either one *H. charithonia* individual homozygous for the inversion (dark blue) or one *H. charithonia* individual

homozygous for the standard haplotype (light blue). The dashed-dotted lines indicate the putative inversion breakpoints according to the local PCA (Fig. S8; Figure 2c).

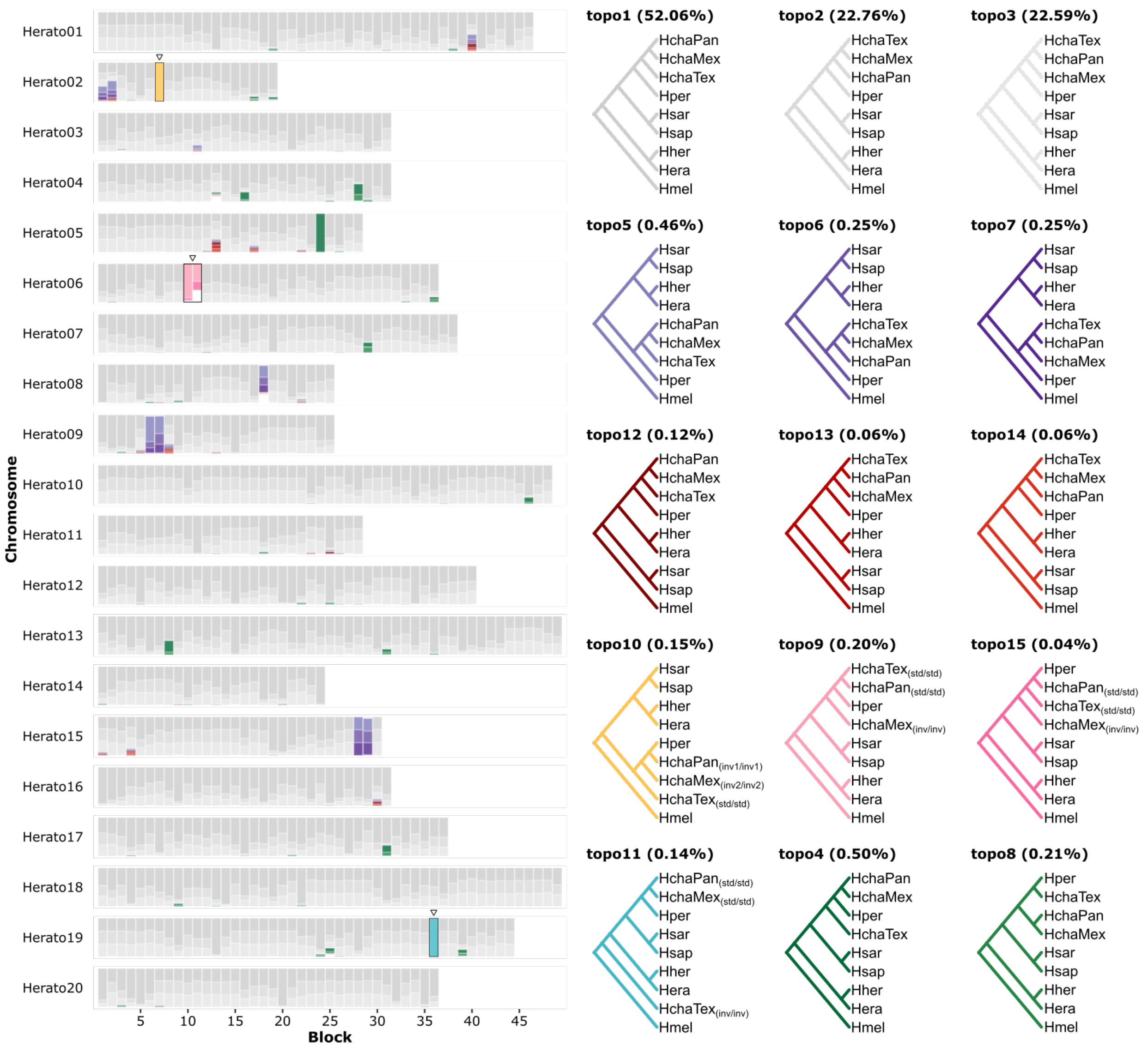

Fig. S17. Estimated species trees across chromosomes under the multispecies coalescent (BPP analyses A01). Each bar represents one block of 100 loci. For each block the posterior probabilities of different trees are given in different colors. Only the 15 most frequent trees are shown, and posterior probabilities of the remaining trees are shown in white. Blocks in inversion regions on chromosomes 2, 6 and 19 are delimited in black and pinpointed by the inverted triangles. Inversion genotypes of *H. charithonia* individuals at each inversion region are given in parentheses (see Supplementary Table 1).

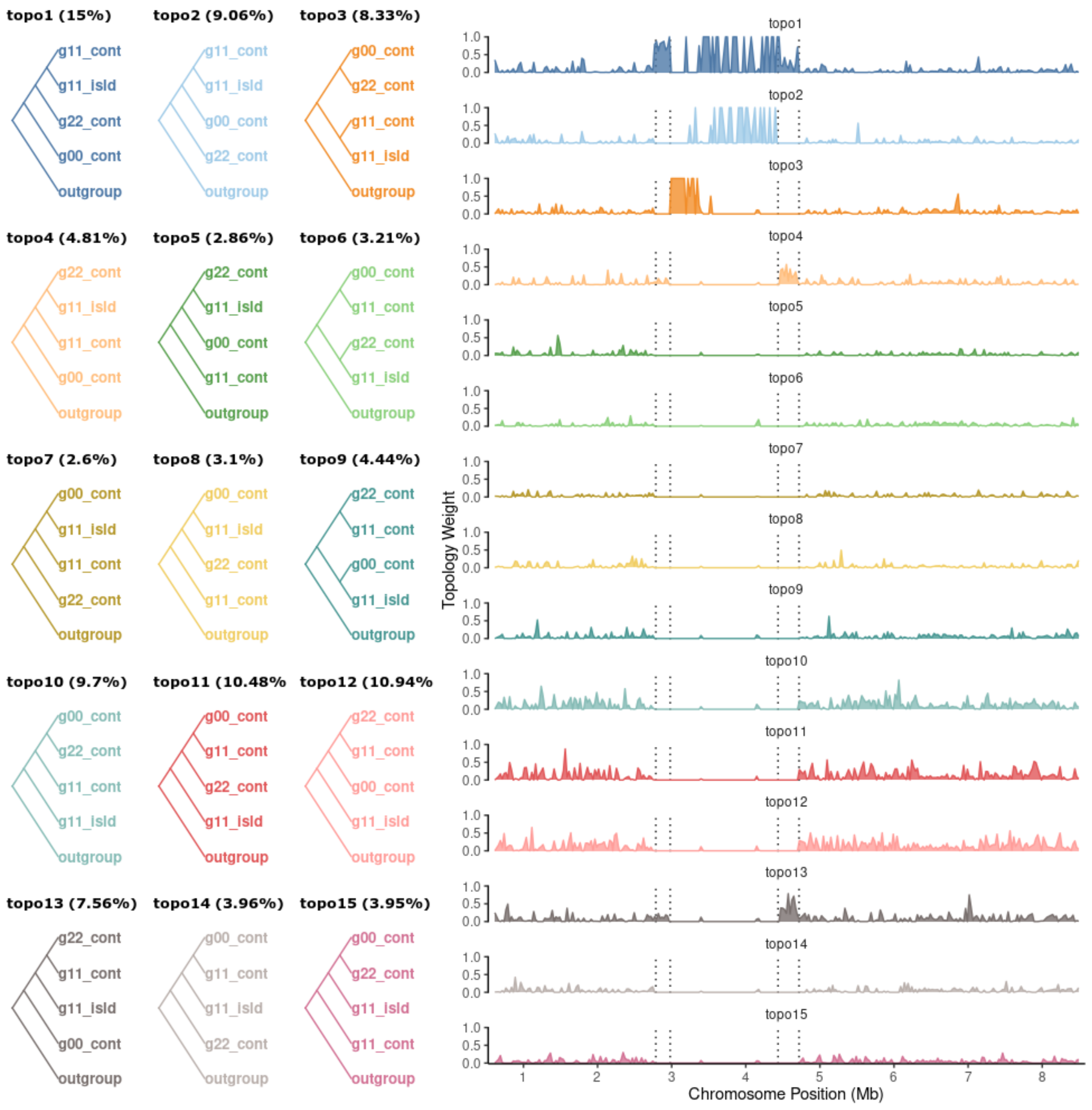

Fig. S18. Relationship between inversion genotypes across chromosome 2. Topology weightings were estimated using Twisst, in non-overlapping 25 kb windows. All 15 possible topologies and their respective average weights across chromosome 2, are indicated on the left. Four groups were considered, three groups including individuals from the Continent homozygous for each of the three inversion haplotypes (g00_cont, g11_cont and g22_cont), and a group with individuals from Jamaica homozygous for the inversion 2 haplotype (g22_isld). In the absence of gene flux, the topology inside the inversion regions should follow the history of divergence of the inversion haplotypes (topo1-3; see Figure 2a). On the other hand, gene flux between inversion haplotypes is expected to result in the grouping of all three populations in the Continent, the island population with the same inversion (g22_isld) as present in the Continent (g22_cont), being basal to that group. The dashed-dotted lines indicate the putative inversion breakpoints according to the local PCA and genome alignments (Fig. S8; Figure 2a).

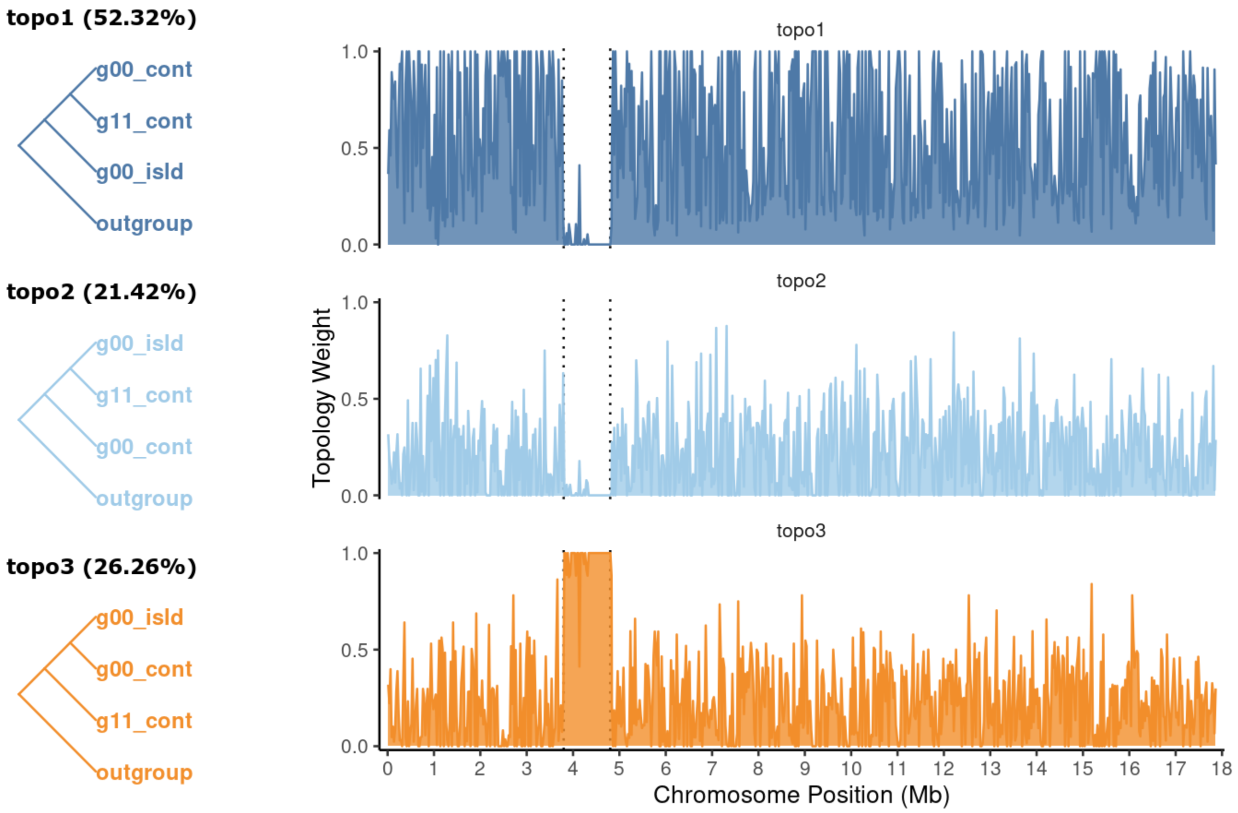

Fig. S19. Relationship between inversion genotypes across chromosome 6. Topology weightings were estimated using Twisst, in non-overlapping 25 kb windows. All 3 possible topologies and their respective average weights across chromosome 6, are indicated on the left. Three groups were considered, two groups including individuals from the Continent homozygous for each of the three inversion haplotypes (g00_cont and g11_cont), and a group with individuals from Jamaica homozygous for non-inverted haplotype (g00_isld). In the absence of gene flux, the two populations carrying the non-inverted haplotype (g00_cont, g00_isld) should be sister taxa (topo3). In the presence of gene flux, we expect populations to group by geographic proximity (topo1). The dashed-dotted lines indicate the putative inversion breakpoints according to the local PCA (Fig. S8; Figure 2b).

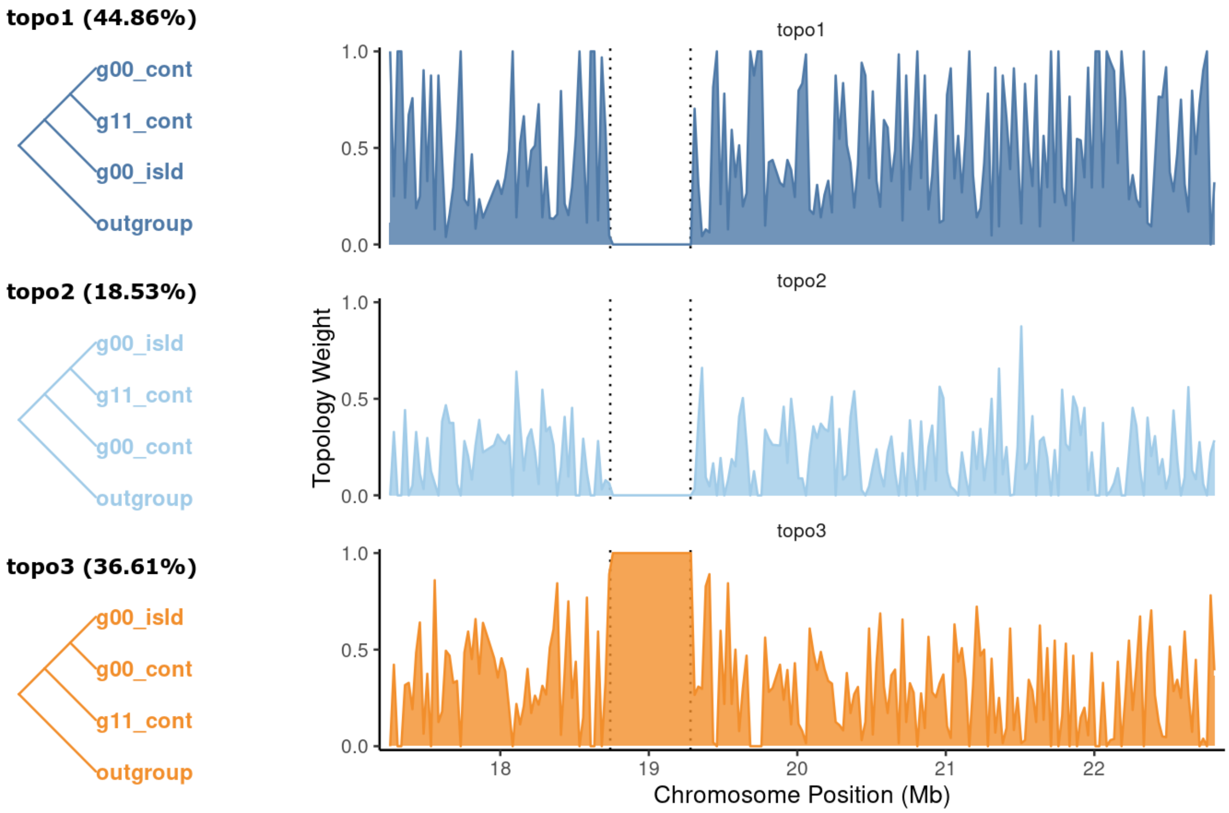

Fig. S20. Relationship across chromosome 19 between inversion genotypes. Topology weightings were estimated using Twisst, in non-overlapping 25 kb windows. All 3 possible topologies and their respective average weights across chromosome 19, are indicated on the left. Three groups were considered, two groups including individuals from the Continent homozygous for each of the three inversion haplotypes (g00_cont and g11_cont), and a group with individuals from Jamaica homozygous for non-inverted haplotype (g00_isld). In the absence of gene flux, the two populations carrying the non-inverted haplotype (g00_cont, g00_isld) should be sister taxa (topo3). In the presence of gene flux, we expect populations to group by geographic proximity (topo1). The dashed-dotted lines indicate the putative inversion breakpoints according to the local PCA (Fig. S8; Figure 2c).

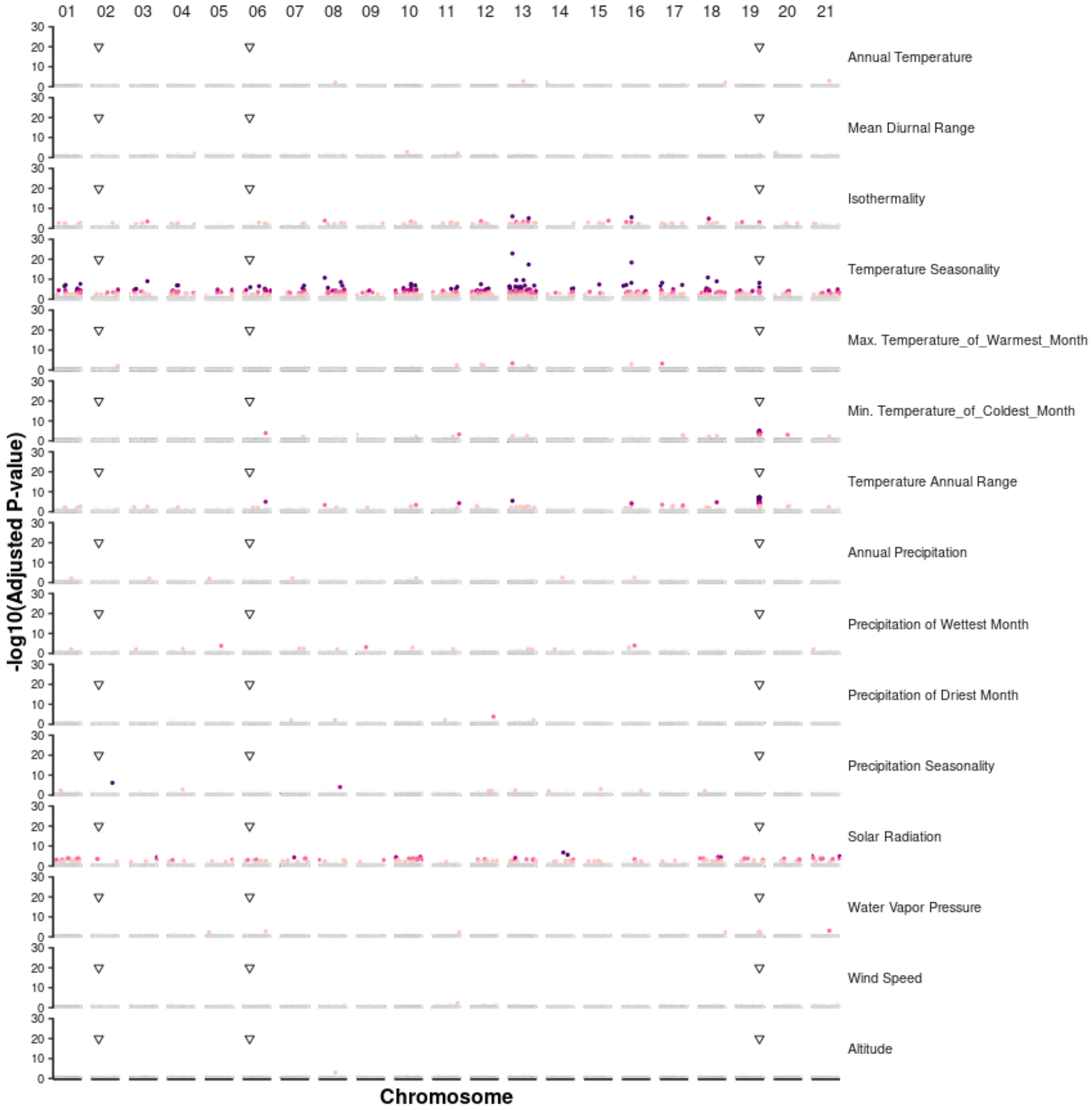

Fig. S21. Genotype-environment associations. The Manhattan plot shows the P values from the latent factor mixed models (LFMM), for K=2. Points are colored according to FDR (purple: <0.00001, dark pink: <0.0001, pink: <0.001, light pink: < 0.01; grey: > 0.001). Inverted triangles represent the inferred location of inversions.

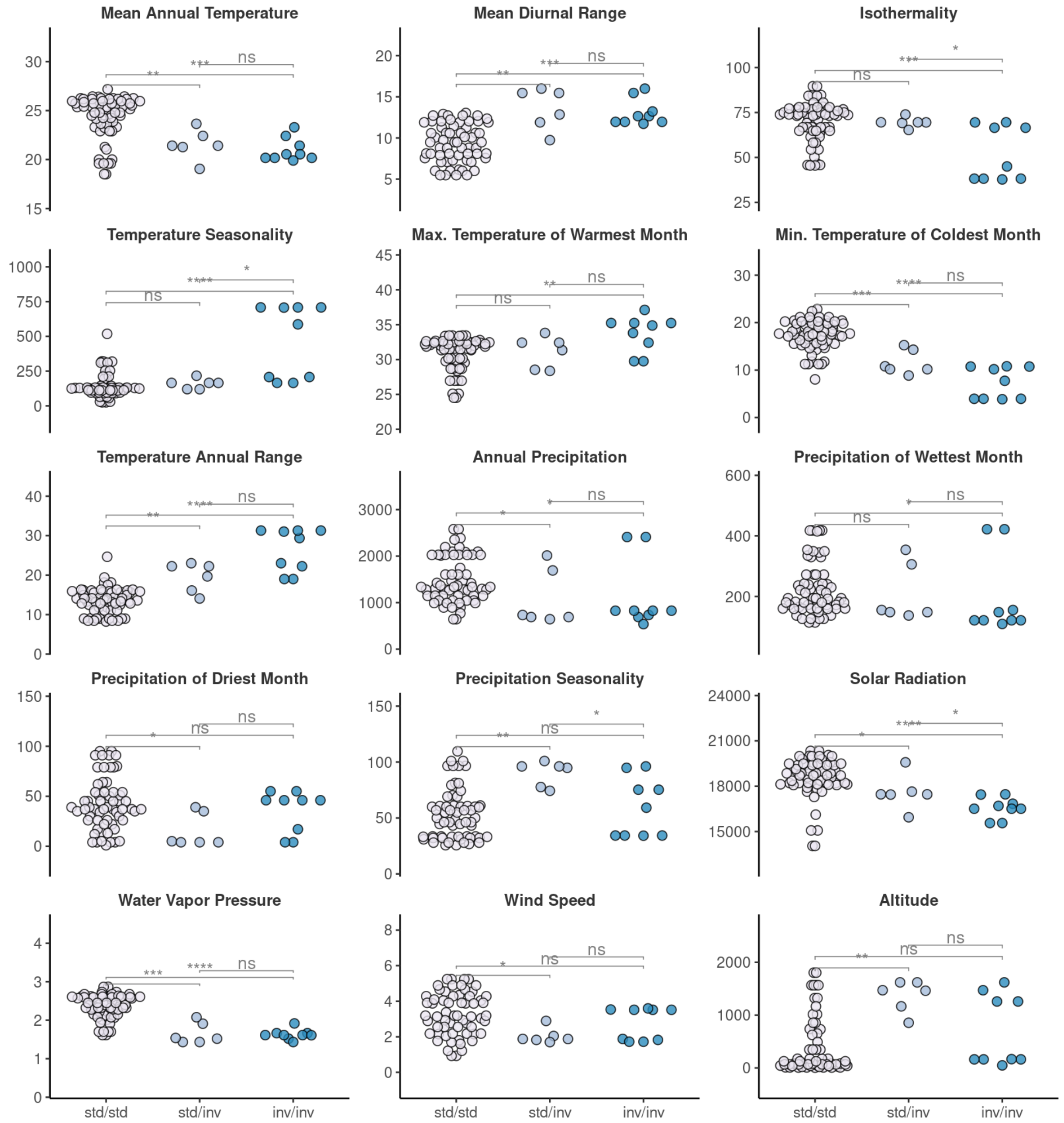

Fig. S22. Environmental conditions by chromosome 19 inversion genotype. Each dot represents one individual, with environmental values at its geographical location shown on the y-axis. Individuals are grouped by their chromosome 19 inversions genotype. Statistical differences among inversion genotypes were assessed with the Wilcoxon signed-rank test (ns: p-value > 0.05; *: p-value ≤ 0.05; **: p-value ≤ 0.01; ***: p-value ≤ 0.001; ***: p-value ≤ 0.0001).

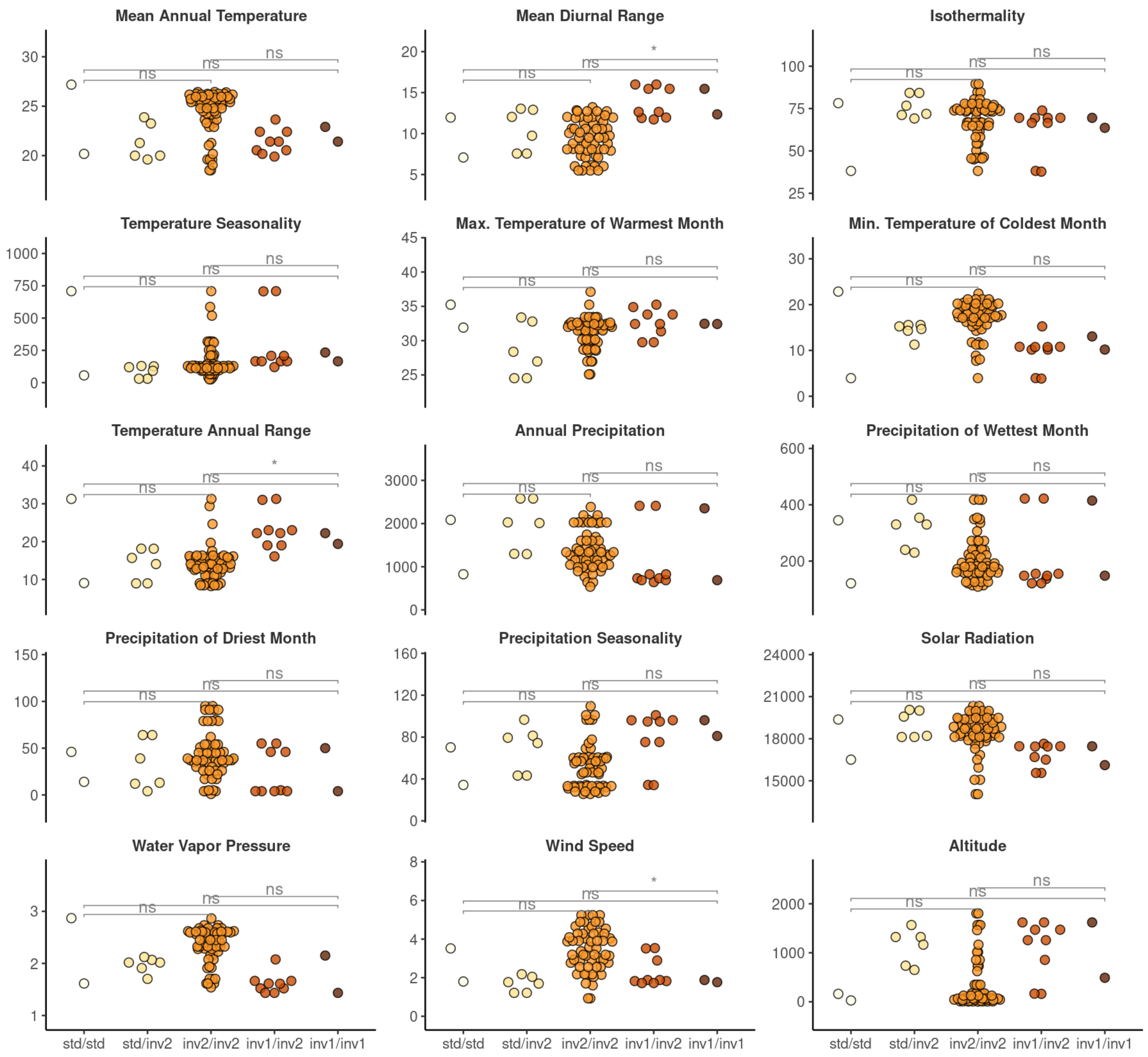

Fig. S23. Environmental conditions by chromosome 2 inversion genotype. Each dot represents one individual, with environmental values at its geographical location shown on the y-axis. Individuals are grouped by their chromosome 2 inversions genotype. Statistical differences among inversion genotypes were assessed with the Wilcoxon signed-rank test (ns: p-value > 0.05; *: p-value ≤ 0.05; **: p-value ≤ 0.01; ***: p-value ≤ 0.001; ***: p-value ≤ 0.0001).

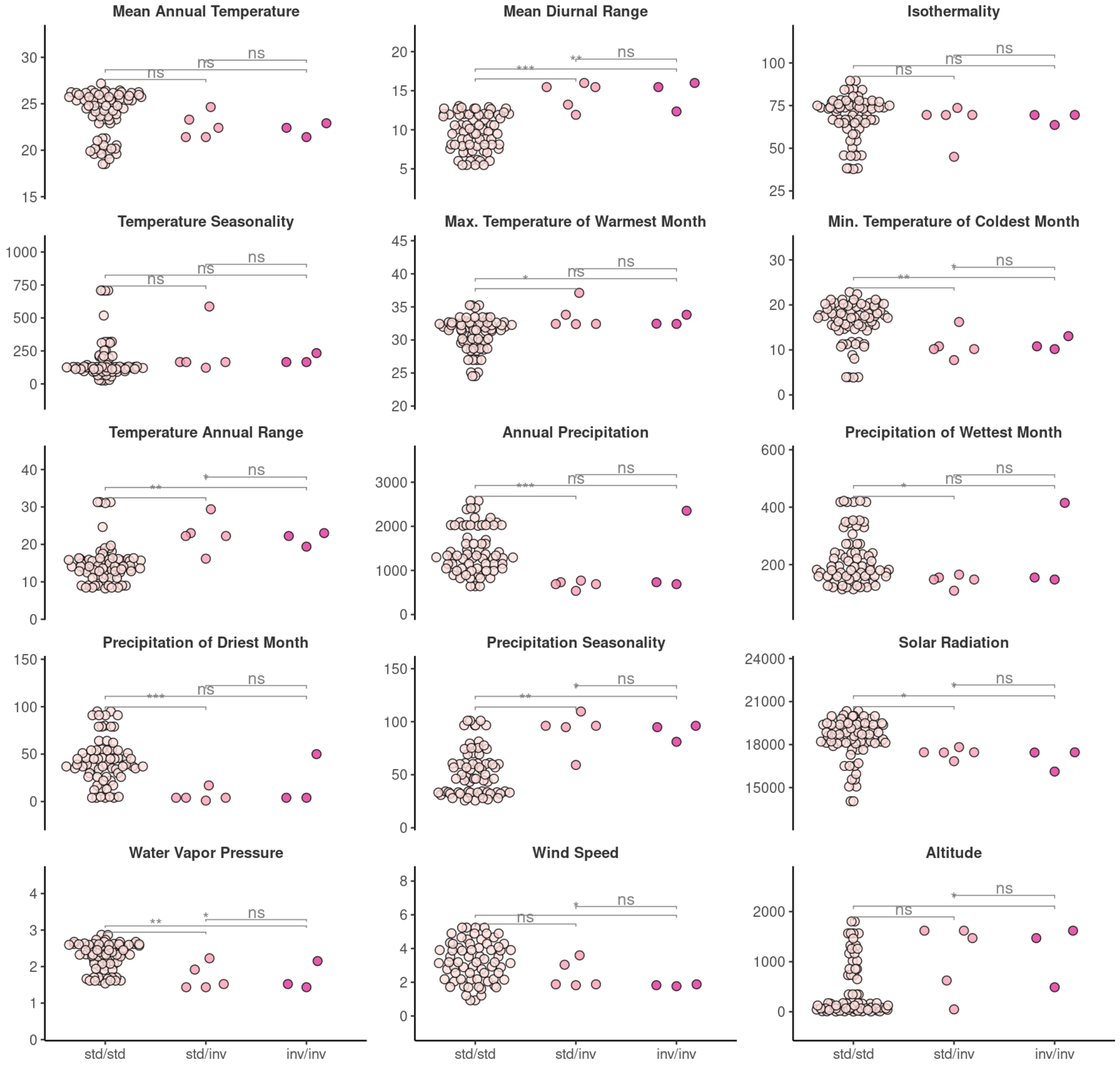

Fig. S24. Environmental conditions by chromosome 6 inversion genotype. Each dot represents one individual, with environmental values at its geographical location shown on the y-axis. Individuals are grouped by their chromosome 6 inversions genotype. Statistical differences among inversion genotypes were assessed with the Wilcoxon signed-rank test (ns: p-value > 0.05; *: p-value ≤ 0.05; **: p-value ≤ 0.01; ***: p-value ≤ 0.001; ***: p-value ≤ 0.0001).

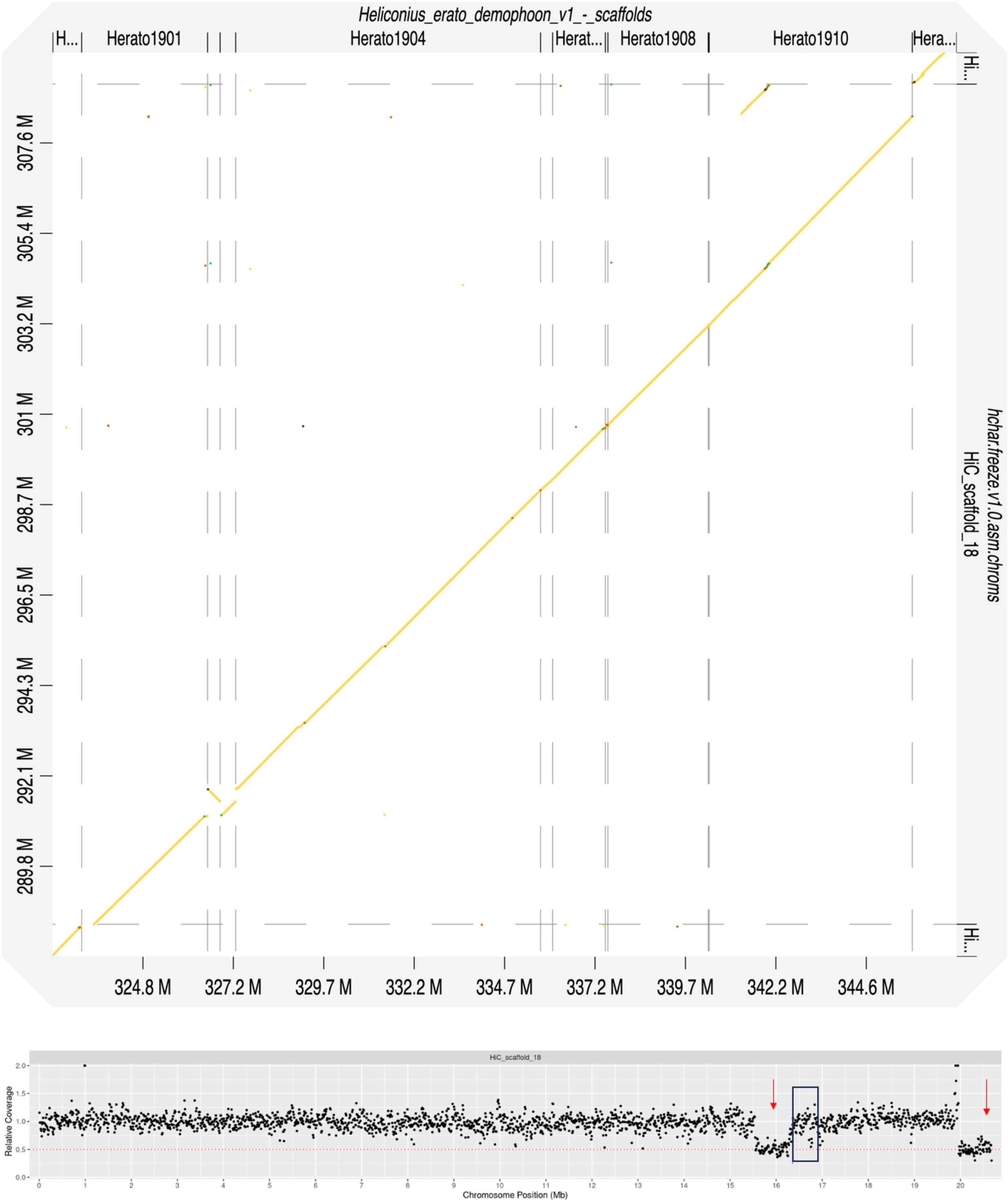

Fig. S25. Texas *H. charithonia* genome mis-assembly on chromosome 19. D-genies genome-genome alignment (top panel) shows two genomic blocks in *H. charithonia* (Texas) genome assembly map to the same region in *H. erato*, immediately adjacent to the putative inversion. Relative coverage along chromosome 19 (bottom panel), shows the two blocks (indicated by arrows), have half of the expected coverage and likely correspond to divergent haplotypes that

were assembled separately. The rectangle indicates the approximate coordinates of the putative inversion.

Table S1. Detailed information of samples included in this study, for which whole genome sequencing data was available or generated. For each sample we provide the following descriptors (when the information is available): *Original Code*: original unique code assigned to the specimen. *SMcode:* unique code assigned to the specimen in this study. *Genus/Species/Subspecies*: Genus, species and subspecies assignment based on phenotype. *Country:* Geographic descriptor of the location from where the sample were collected. *Location Code*: unique location code specifying the population to which each specimen was assigned*.* *Population Code:* unique population code specifying the population to which each specimen was assigned. *Latitude/Longitude:* Geographic coordinates of the location from where the sample was collected. When exact coordinates were not available, approximate coordinates were chosen based on locality. *Origin: Molecular Sex:* individuals sex determined based on the median coverage of the Z-chromosome relative to median coverage of autosomes: ~0.5 for females (F) and ~1 for males (M). *Median Coverage:* median coverage calculated in 25-kb windows. *Reference:* Study where the data was originally published. *Sample Accession:* NCBI SRA BioSample accession identifiers. Samples sequenced in this study are highlighted in bold. *mtDNA cluster*: haplogroup based on the mtDNA phylogeny. *Inversion Genotype:* Genotypes at inversion regions as determined by PCA.

Table S2. Parameter estimates from inversion regions (and collinear lineages, for reference) using the multispecies coalescent (MSC) approach implemented in BPP. Both raw values and scaled parameters (assuming a mutation rate of 2.9 x 10-9 mutations/site/generation) are presented.

Table S3. Inversion breakpoints and genes within inversions. Genes coordinates are based on the *H. e. demophoon* reference genome annotation. Orthologous genes in *Drosophila melanogaster* determined based on BLAST best hit (BLAST scores are provided).

Table S4. No evidence of gene flux between inversion haplotypes. Parameter estimates under the Strict Isolation (DIV) and Isolation-with-Migration (IM) – assuming migration from A to B (IM_AB) or B to A (IM_BA) – inside inversions using gIMble. Scaled parameters are presented assuming a mutation rate of 2.9 x 10-9 mutations/site/generation. Population codes follow inversion genotypes as shown in Table S1.

Table S5. Climatic variables obtained from the WorldClim database (at 2.5 arc-minutes – ca. 4.6 km2 resolution) extracted for each individual.
